# Deletion of *Slc9a1* in *Cx3cr1*^+^ cells stimulated microglial subcluster CREB1 signaling and microglia-oligodendrocyte crosstalk

**DOI:** 10.1186/s12974-024-03065-z

**Published:** 2024-03-20

**Authors:** Shanshan Song, Helena Oft, Shamseldin Metwally, Satya Paruchuri, John Bielanin, Victoria Fiesler, Chaim Sneiderman, Gary Kohanbash, Dandan Sun

**Affiliations:** 1 Department of Neurology, University of Pittsburgh, 3501 Fifth Avenue, Pittsburgh, PA 15213 USA; 2 Pittsburgh Institute for Neurodegenerative Disorders, University of Pittsburgh, Pittsburgh, PA USA; 3 Department of Neurological Surgery, University of Pittsburgh, Pittsburgh, PA USA; 4 Veterans Affairs Pittsburgh Health Care System, Pittsburgh, PA 15213 USA

**Keywords:** Microglia, Na^+^/H^+^ exchanger isoform-1 (NHE1), Oligodendrocytes, Single cell RNAseq, Stroke, White matter myelination

## Abstract

Microglial Na/H exchanger-1 (NHE1) protein, encoded by *Slc9a1*, plays a role in white matter demyelination of ischemic stroke brains. To explore underlying mechanisms, we conducted single cell RNA-seq transcriptome analysis in conditional *Slc9a1* knockout (cKO) and wild-type (WT) mouse white matter tissues at 3 days post-stroke. Compared to WT, *Nhe1* cKO brains expanded a microglial subgroup with elevated transcription of white matter myelination genes including *Spp1*, *Lgals3*, *Gpnmb*, and *Fabp5*. This subgroup also exhibited more acidic pH_i_ and significantly upregulated CREB signaling detected by ingenuity pathway analysis and flow cytometry. Moreover, the *Nhe1* cKO white matter tissues showed enrichment of a corresponding oligodendrocyte subgroup, with pro-phagocytosis and lactate shuffling gene expression, where activated CREB signaling is a likely upstream regulator. These findings demonstrate that attenuation of NHE1-mediated H^+^ extrusion acidifies microglia/macrophage and may underlie the stimulation of CREB1 signaling, giving rise to restorative microglia-oligodendrocyte interactions for remyelination.

**Supplementary Information:**

The online version contains supplementary material available at 10.1186/s12974-024-03065-z.

## Introduction

Microglia regulate white matter myelination in both developing and post-injury brains [1, 2] and present a promising target for white matter repair [3]. However, microglial cells are highly plastic and can acquire differential phenotypes across a range of spectrum [3]. New single cell RNA-sequencing (scRNA-seq) studies have revealed the presence of various microglial subclusters within the brain after ischemic stroke, each displaying distinct transcriptomic profiles [4–6]. Subclusters associated with resting microglia, proliferative microglia, or anti-inflammatory microglia were identified based on their expression patterns of homeostatic, inflammatory, or repair-promoting genes [4–6]. Microglia can exert damaging effects post-stroke, by releasing inflammatory cytokines, hindering oligodendrocyte maturation, and promoting axonal degeneration [7–9]. Alternatively, they can regulate adaptive functions by releasing restorative cytokines and growth factors, clearing tissue debris through phagocytosis, and promoting remyelination [8, 10, 11]. The balance between detrimental and restorative state microglial subclusters is critical for initiating oligodendrocyte differentiation and the onset of remyelination [10]. However, how to modulate specific microglial transition to anti-inflammatory and myelin-supporting functions is not well understood. A precise dissection of the specific roles of each cell type and their interactions facilitating white matter injury and myelin repair is also urgently warranted.

The Na^+^/H^+^ exchanger-1 (NHE1) serves as a key regulator for intracellular pH (pH_i_) in microglia by extruding H^+^ ions in exchange of Na^+^ influx [12, 13]. In recent studies, we reported that selective deletion of microglial *Nhe1* in the *Cx3cr1-Cre*^*ER*±^*;Nhe1*^*flox/flox*^ (cKO) mice reduced proinflammatory responses at 3 days post-stroke [12] and improved oligodendrogenesis along with remyelination tested at 3, 14, and 28 days post-stroke in the *Nhe1* cKO mice [13]. We detected enhanced microglial oxidative phosphorylation and phagocytosis of microglial cells in the *Nhe1* cKO mice at 3 days post-stroke [13]. To further investigate the underlying mechanisms for these phenotypic changes, and to reveal whether the selective deletion of *Nhe1* in cKO brains causes uniform alterations among different microglial subpopulations, and whether it influences their functions and interactions with other cell types, we conducted scRNAseq transcriptome analysis at 3 days post-stroke timepoint to explore mechanisms between microglia-oligodendrocyte interactions. Our analysis revealed a total of 24 transcriptionally distinct cell populations within the white matter tissues, encompassing various cell types including oligodendrocytes, microglia, and astrocytes, etc. Notably, we identified 5 subpopulations for microglia (MG) and 5 for oligodendrocytes (OL), where MG3 and OL5 subpopulations were expanded in the *Nhe1* cKO white matter tissues. These subpopulations exhibited a unique genetic signature characterized by elevated lipid metabolism, enhanced phagocytosis, improved lysosome functions, and an array of myelin-supporting genes, as well as genes associated with lactate shuttling functions. In summary, our findings highlight the critical role of the pH-regulatory NHE1 protein in modulating of microglial phenotypes and microglia-oligodendrocyte interactions.

## Results

### scRNAseq identified various cell types in post-stroke white matter tissues

**Fig. 1:**
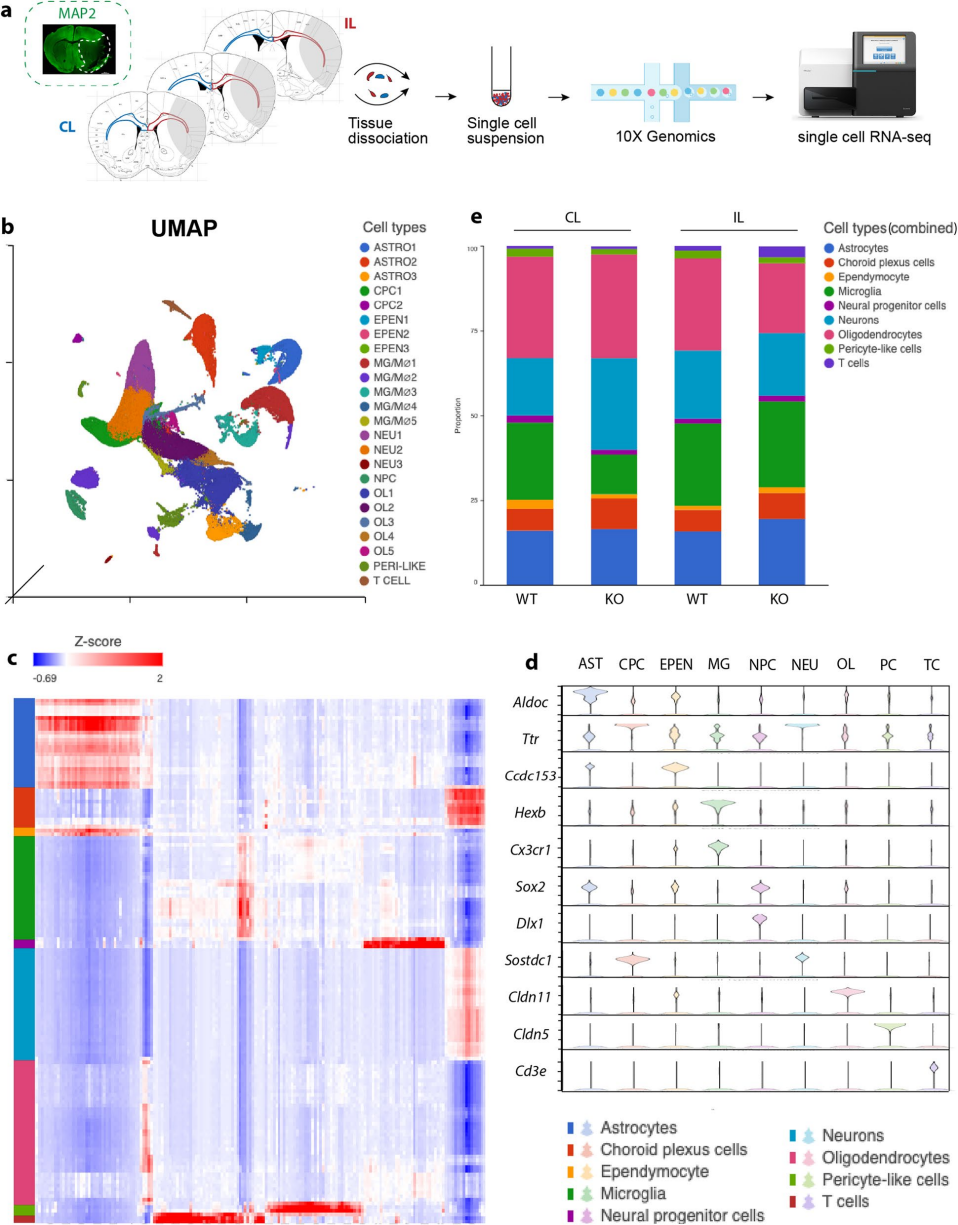
scRNAseq of white matter tissues identified various cell types. **a** Illustration of sample collection sites with MAP2 staining and experimental protocol of single cell isolation from contralateral (CL) and ipsilateral (IL) white matter tissues. **b** UMAP of all cell types from CL and IL hemispheres of WT and cKO white matter tissues. **c** Heatmap showing separation of cell types. **d** Violin plots of signature marker genes of each cell type. **e** Proportion of each cell type in CL or IL hemispheres of WT and cKO white matter tissues

White matter consists of axonal fibers and various types of glial cells, including the myelin-secreting OLs, OL progenitor cells (OPCs), microglia, and astrocytes, etc. To dissect the cellular complexity and their communications, and to avoid bias of using cell surface markers that may be differentially expressed in specific populations, we isolated the entire white matter tissues (corpus callosum and external capsules) from each hemisphere of the WT or cKO brains at 3 days post-stroke and performed scRNAseq with 10X Genomics (Fig. 1a). Our previous study of TTC staining and MAP2 immunostaining revealed infarction encompassing cortex and white matter tissues in WT and cKO brains [12]. Therefore, microglia within the area of infarction were also included in our scRNAseq sampling and analysis. After quality control, a total of 152,770 cells from 12 samples (3 biological replicates per group) were used for downstream analyses. Unique molecular identifiers (UMI) counts, detected features, and mitochondrial percentages were shown in Additional file 1: Figure S1a.

Unsupervised clustering with UMAP identified 24 transcriptionally distinct cell clusters, which were then annotated into 9 cell types based on their overrepresented gene markers (Fig. 1b–d). Specifically, we identified 5 oligodendrocyte subclusters (OL1-5, with high level of *Cldn11*), 5 microglial subclusters (MG1-5, with high level of *Hexb*), 3 astrocyte subclusters (ASTRO1-3, with high level of *Aldoc*), 3 ependymal subclusters (EPEN1-3, with high level of *Ccdc153*), 3 neuron subclusters (NEU1-3, with high level of *Sostdc1*), 2 choroid plexus subclusters (CPC1-2, with high level of *Ttr*), 1 neuron progenitor cell cluster (NPC, with high level of *Sox2*), 1 pericyte-like cell cluster (with high level of *Cldn5*), and 1T-cell cluster (with high level of *Cd3e*) (Fig. 1b–d). White matter tissues from both the non-stroke contralateral (CL) hemispheres and the stroke ipsilateral (IL) hemispheres from WT and cKO mice contained a complete panel of all cell types, and the proportion of most aggregated cell type remained relatively unchanged (Fig. 1e). Regarding the seemingly decreased microglia population in the CL hemisphere of cKO brains, we conducted additional analysis by excluding the potential contaminating cell types in the white matter tracts, notably neurons and various cell types from choroid plexus tissues (choroid plexus cells, ependymocytes, pericyte-like cells, etc.). cKO CL showed decreased percentage of microglial cells, however, the absolute cell counts were comparable between WT and cKO (both CL and IL hemispheres) (Additional file 1: Figure S1b). We speculate that this was due to the significantly increased preservation of oligodendrocyte counts in the cKO CL hemisphere, thus yielding a reduced percentage of microglial cells.

Overall, we identified various cell types in the white matter tissues after stroke. Unlike gray matter, where stroke induces significant loss of neurons and infiltration of immune cells [14], the post-stroke white matter did not show such profound alterations in cell type compositions. However, significant differential changes in the subgroups of microglia and oligodendrocyte were found as described below.

### *Nhe1* cKO white matter selectively expanded MG3 with increased genes for lipid metabolism, phagocytosis, lysosomal functions, and myelin-supporting functions

**Fig. 2:**
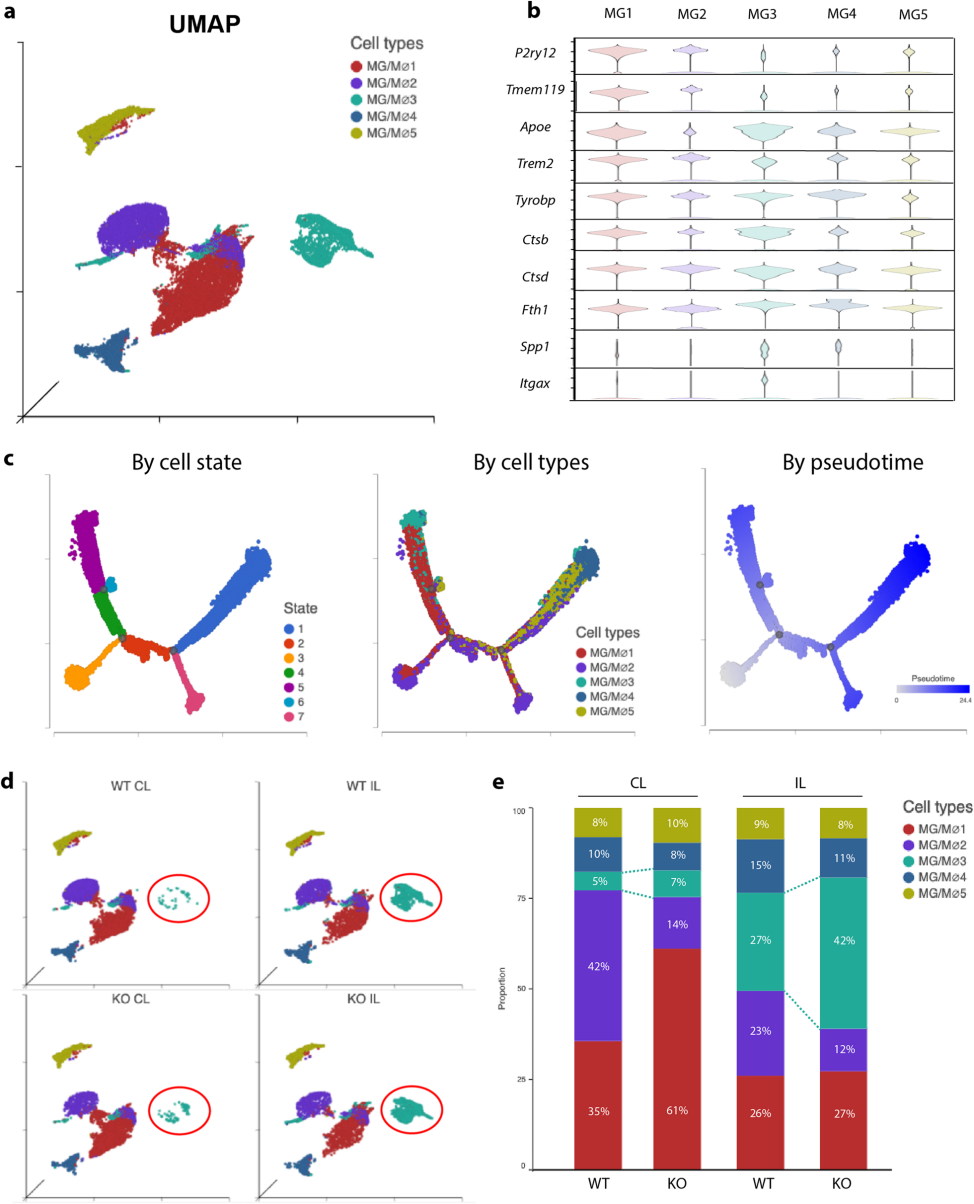
*Nhe1* cKO specifically expanded a microglial subcluster with increased lipid metabolism, phagocytosis and lysosomal functions in post-stroke white matter tissues. **a** UMAP of microglial subtypes from CL and IL hemispheres of WT and cKO white matter tissues. **b** Violin plots of signature genes for homeostatic microglia and disease-associated microglia (DAM) in each microglial subtype. **c** Trajectory analysis of microglial cells annotated by cell state, microglial subtype, or developmental pseudotime. **d** Split view of UMAP of microglial cells in CL or IL hemispheres of WT and cKO white matter tissues. **e** Proportion of microglial subtypes in CL or IL hemispheres of WT and cKO white matter tissues

**Fig. 3:**
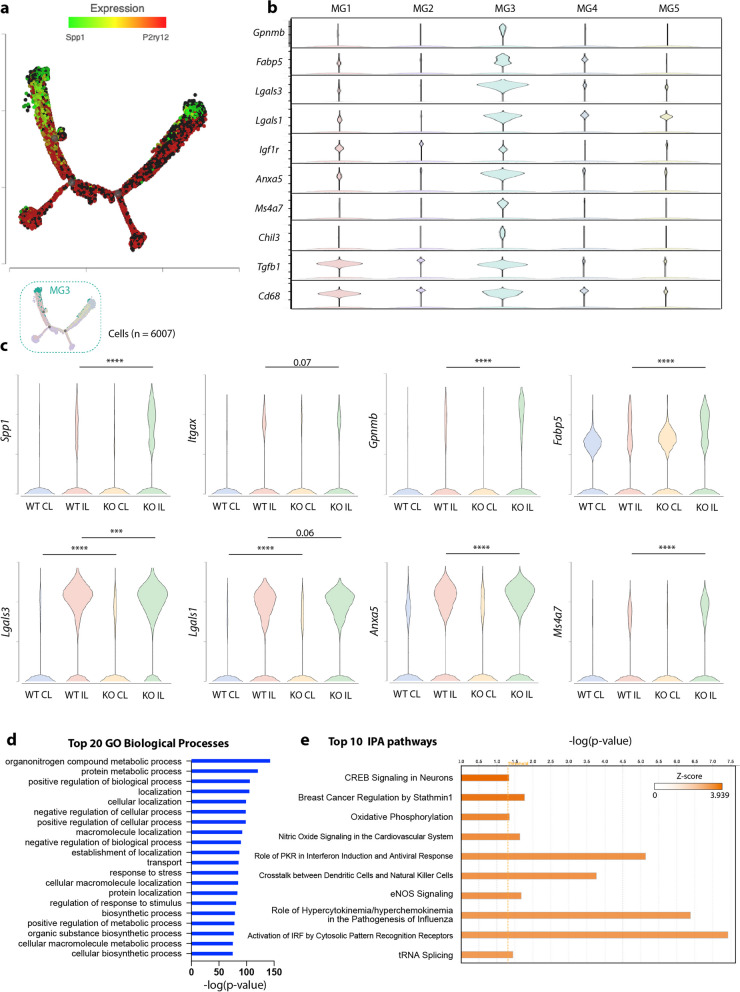
The MG3 subcluster specifically increased genes for myelin-supporting functions in the *Nhe1* cKO white matter tissues after stroke. **a** Trajectory analysis showing differential microglial states annotated by *Spp1* and *P2ry12*. **b** Violin plots of myelin-supporting genes identified MG3 as the microglial subgroup with myelin-supporting features. **c** *Nhe1* cKO exhibited increased myelin-supporting gene expressions in MG3 subtype in white matter tissues of IL hemisphere compared to WT. **d** Gene Ontology (GO) analysis for the top 20 biological processes of differentially expressed genes (DEGs) from MG3 subpopulation. **e** Ingenuine Pathway Analysis (IPA) of DEGs of MG3 subpopulation, in descending order by z score

In our scRNAseq dataset, all 5 transcriptionally distinct microglial subclusters (MG1-5, Fig. 2a, Additional file 1: Figure S2a) expressed high levels of microglial specific marker *Hexb* (Additional file 1: Figure S2b). MG1 and MG2 represent the homeostatic resting states with high levels of *P2ry12* and *Tmem119* (Fig. 2b), while MG3-5 likely represent microglial activated states considering the drastically reduced expressions of *P2ry12* and *Tmem119* upon injury [15] (Fig. 2b). Trajectory analysis with MG1 and MG2 as the roots illustrated 2 differential trajectories of microglial development—one arm with MG5 as a transition state and MG4 as the end state, and the other arm with a significant amount of preserved homeostatic MG1, and MG3 as the end state (Fig. 2c), suggesting a developmental similarity of MG3 to the homeostatic microglia. Among MG1-5 microglial subclusters, ischemic stroke most significantly increased the MG3 subgroup, compared to the non-stroke CL hemispheres (Fig. 2d, e). Interestingly, the MG3 subgroup in the IL white matter of *Nhe1* cKO brains expanded by 1.5-fold of that in the WT, and by a small increase in the CL hemisphere as well (Fig. 2e). The MG3 subgroup displayed high expressions of genes involved in lipid metabolism, phagocytosis, and lysosomal functions, such as *Apoe, Trem2, Tyrobp, Ctsb, Ctsd*, etc. (Fig. 2b), resembling both microglia during early development where they have high plasticity [16], and reported disease-associated microglial (DAM) or the neurodegeneration-associated microglial (MGnD) profiles [17–19]. Compared to MG4 and MG5, the MG3 subpopulation was characterized by an almost exclusively high expression of a panel of genes involved in myelin-supporting functions, such as *Spp1, Itgax, Gpnmb, Fabp5, Lgals3, Lgals1, Igf1r, Anxa5, and Ms4a7*, etc. (Figs. 2b, 3a, b). This signature aligns with the reported “axon tract-associated microglia” (ATM) [20]. In addition, MG3 also expressed elevated expressions of *Chil3* (encoding Ym1), and displayed high levels of *Tgfb1* and *Cd68* similar to the homeostatic MG1 (Fig. 3b), indicating higher anti-inflammatory response, growth factor release, and microglial phagocytic activity [12]. Interestingly, *Nhe1* cKO white matter tissues further heightened this expression pattern in the MG3 subgroup compared to WT animals (Fig. 3c). Gene Ontology (GO) analysis revealed metabolic processes were the most significantly altered biological processes, while IPA results implicated CREB signaling and oxidative phosphorylation among the top upregulated pathways in MG3 of *Nhe1* cKO white matter (Fig. 3d, e, Additional file 1: Figure S3). These novel findings suggest that *Nhe1* cKO specifically expanded a microglial subcluster with increased lipid metabolism, phagocytosis, lysosomal functions, and myelin-supporting functions in the white matter tissues, and upregulated pathways involving CREB signaling and energy metabolism.

### Deletion of microglial *Nhe1* acidified microglia with activated CREB1 signaling in ischemic brains

**Fig. 4:**
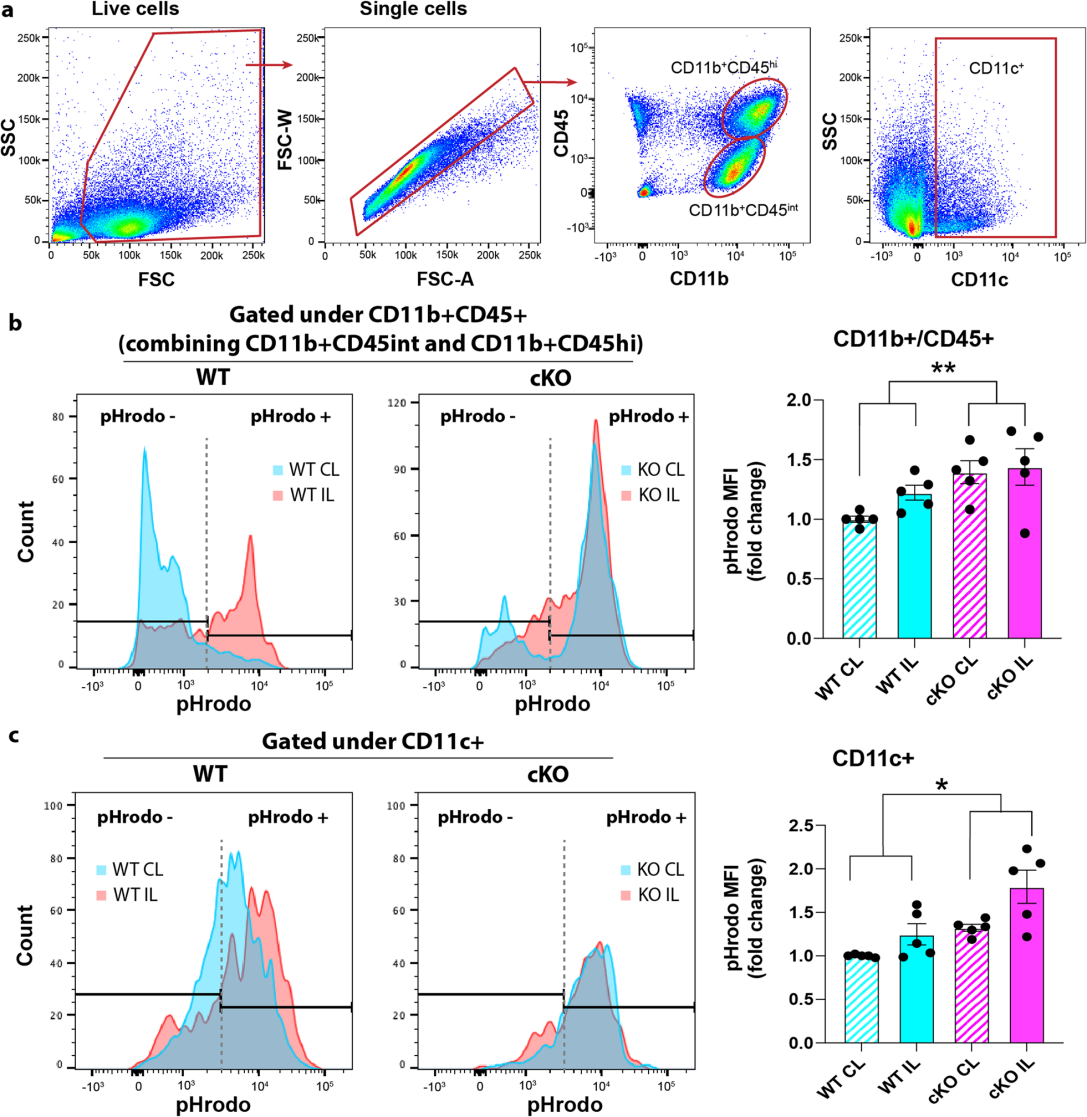
*Nhe1* cKO acidified microglia/macrophages in ischemic brains. **a** Representative gating strategies of CD11b^+^CD45^int^ and CD11b^+^CD45^hi^ microglia/macrophages. **b**, **c** Mean fluorescent intensity (MFI) of pHrodo^+^ cells within CD11b^+^CD45^+^ (combining CD11b^+^CD45^int^ and CD11b^+^CD45^hi^) or CD11c^+^ microglia/macrophage populations in contralateral (CL) and ipsilateral (IL) hemispheres of WT and cKO mice. Data are expressed as mean ± SEM, N = 5. * p < 0.05, ** p < 0.01

We speculate that altered pH_i_ homeostasis underlies these transcriptional changes in the *Nhe1* cKO microglia. It has been reported that CREB signaling activation promotes glucose metabolism in acidified pH_i_ conditions [21]. Therefore, to study the pH_i_ changes in WT or *Nhe1* cKO microglia, we conducted flow cytometry with a novel pH indicator pHrodo whose fluorescent intensity is inversely correlated with pH_i_ [22] (Fig. 4a). Brain-resident microglia are traditionally characterized as CD11b^+^CD45^int^ population and peripheral macrophages as CD11b^+^CD45^hi^ population [23] (Fig. 4a). However, after ischemic stroke or other inflammatory conditions, microglia can upregulate CD45 expression and the overlapping signature marker expressions are indistinguishable from the CD11b^+^CD45^hi^ macrophages [24–27]. Initially, we analyzed pH_i_ in the combined CD11b^+^CD45^+^ (CD11b^+^CD45^int^ and CD11b^+^CD45^hi^) population as “microglia/macrophages”. As CD11c (encoded by Itgax) is almost exclusively expressed in the MG3 subgroup (Fig. 2b), we were able to dissect MG3 using CD11c labeling under flow cytometry, which could not be achieved otherwise in such a small population [28]. No differences were detected in pH_i_ in either CD11b^+^/CD45^+^ or CD11c^+^ populations between sham-operated WT or cKO mice (Additional file 1: Figure S4a). Stroke induced an increased pHrodo fluorescent intensity in the CD11b^+^/CD45^+^ microglia/macrophages of the IL hemisphere, compared to CL hemisphere from the WT brains (Fig. 4b), likely due to stroke-induced acidosis. Interestingly, the CD11b^+^/CD45^+^ microglia/macrophages in both hemispheres of the cKO brains displayed higher intensity of pHrodo relative to WT tissues (Fig. 4b), suggesting that deletion of *Nhe1* gene further acidified the cKO microglia/macrophages after abolishment of NHE1-mediated H^+^ extrusion activity. Interestingly, the CD11c^+^ MG3 also showed significantly increased fluorescent intensity of pHrodo in the *Nhe1* cKO brains, compared to WT (Fig. 4c), consistent with our hypothesis that cell acidification may mediate the gene expression changes observed in this group.

It has been shown that increased microglial acidosis reverses neurodegenerative pathology in Alzheimer’s disease due to more effective lysing of the engulfed content for effective clearance and recycling [29]. Our results show that prevention of H^+^ extrusion in *Nhe1* cKO white matter acidifies microglia/macrophages, which could alter microglial metabolism and lead to signaling transcriptome changes.

**Fig. 5:**
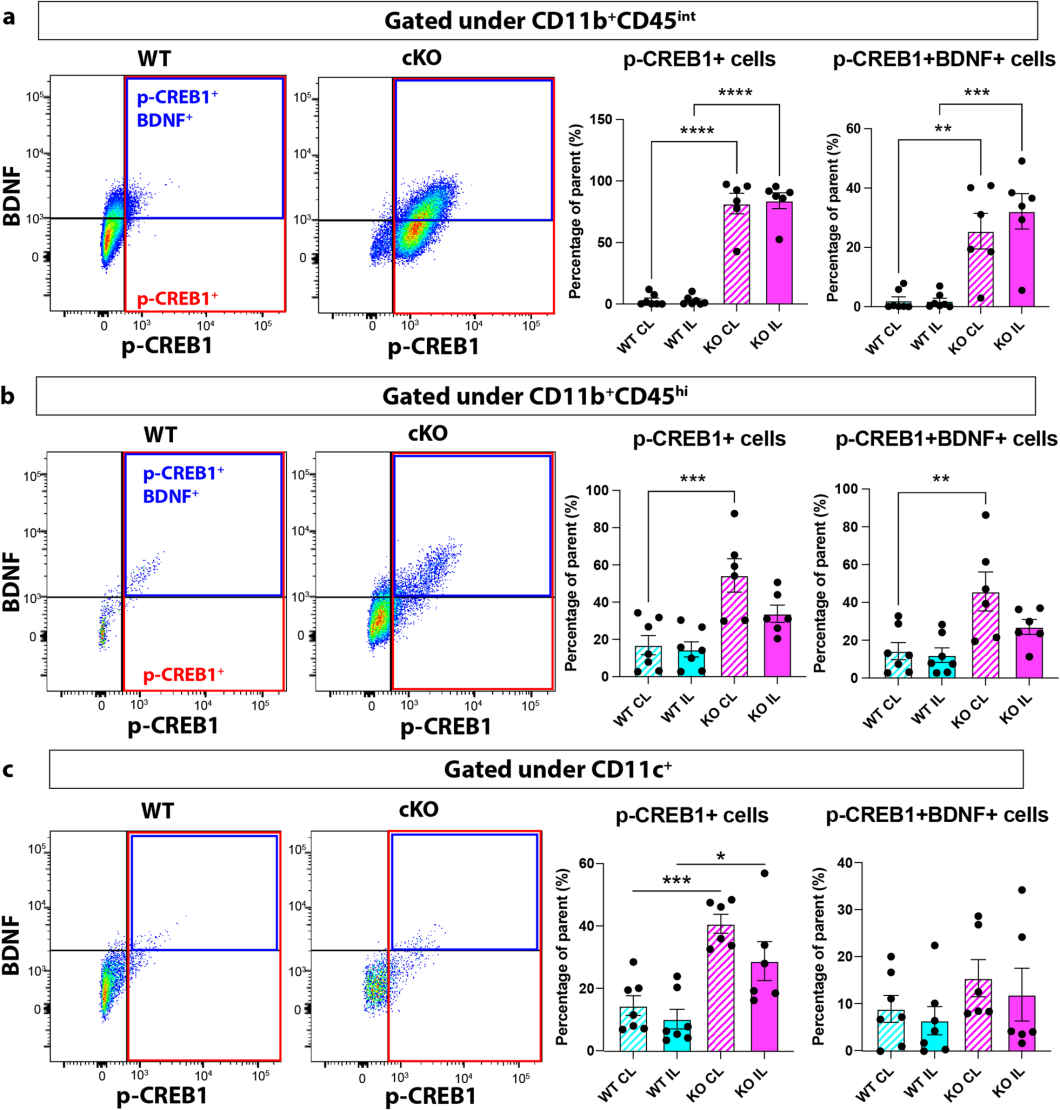
*Nhe1* cKO increased CREB1 activation in microglia/macrophages after stroke. Representative gating strategies and analysis of p-CREB1^+^ and p-CREB1^+^BDNF^+^ cell counts within the parent **a** CD11b^+^CD45^int^ microglia, **b** CD11b^+^CD45^hi^ microglia/macrophages, and **c** CD11c^+^ microglia/macrophage populations. Data are expressed as mean ± SEM, N = 7. * p < 0.05, ** p < 0.01, *** p < 0.001, **** p < 0.0001

We further investigated CREB signaling activation in microglial cells in both WT and cKO brains. CREB transcription factors are required for the early induction of all the major BDNF transcripts [30], and the activation of CREB-induced BDNF secretion is crucial for cortical circuit plasticity and functional recovery after stroke in mice [31, 32]. Interestingly, both CD11b^+^CD45^int^ microglia and CD11b^+^CD45^hi^ microglia/macrophages showed significant increases in p-CREB1^+^and p-CREB1^+^ p-CREB1^+^ expressions in the cKO brains, respectively (Fig. 5a, b). Stroke induced a 61% increase of p-CREB expressions in the combined CD11b^+^/CD45^+^ microglia/macrophage populations in the WT IL compared to their non-stroke CL hemispheres, and a 2.07-fold increase of BDNF expressions via intracellular staining within these microglia/macrophages post-stroke (Additional file 1: Figure S4c). Compared to WT, *Nhe1* cKO brains further boosted a 9- to 16-fold increase in the expression of activated p-CREB1 protein in the combined CD11b^+^/CD45^+^ microglia/macrophages, and significantly increased expression of BDNF within these p-CREB1^+^/CD11b^+^/CD45^+^ microglia/macrophages (Additional file 1: Figure S4d). The CD11c^+^ MG3 population also exhibited a significant elevation of p-CREB1 in *Nhe1* cKO brains, compared to WT (Fig. 5c). However, BDNF secretion within these p-CREB1^+^/CD11c^+^ cells were unchanged between the two groups (Fig. 5c), indicating that additional downstream pathways may be involved in the CREB signaling, such as PEPCK/G6Pase for glucose metabolism, STAT3 transcription activation, Cyclin D1/A for cell survival, etc. [33]. Further investigation is warranted to identify the specific downstream pathway after the CREB signaling activation in these microglia. These data corroborated our scRNAseq results and strongly suggest that selective deletion of microglial *Nhe1* elevated CREB signaling activation, which likely promoted the restorative microglia-oligodendrocyte crosstalk in white matter repair.

### Post-stroke *Nhe1* cKO white matter tissues specifically elevated an oligodendrocyte subcluster with phagocytosis and lactate shuttling functions

**Fig. 6:**
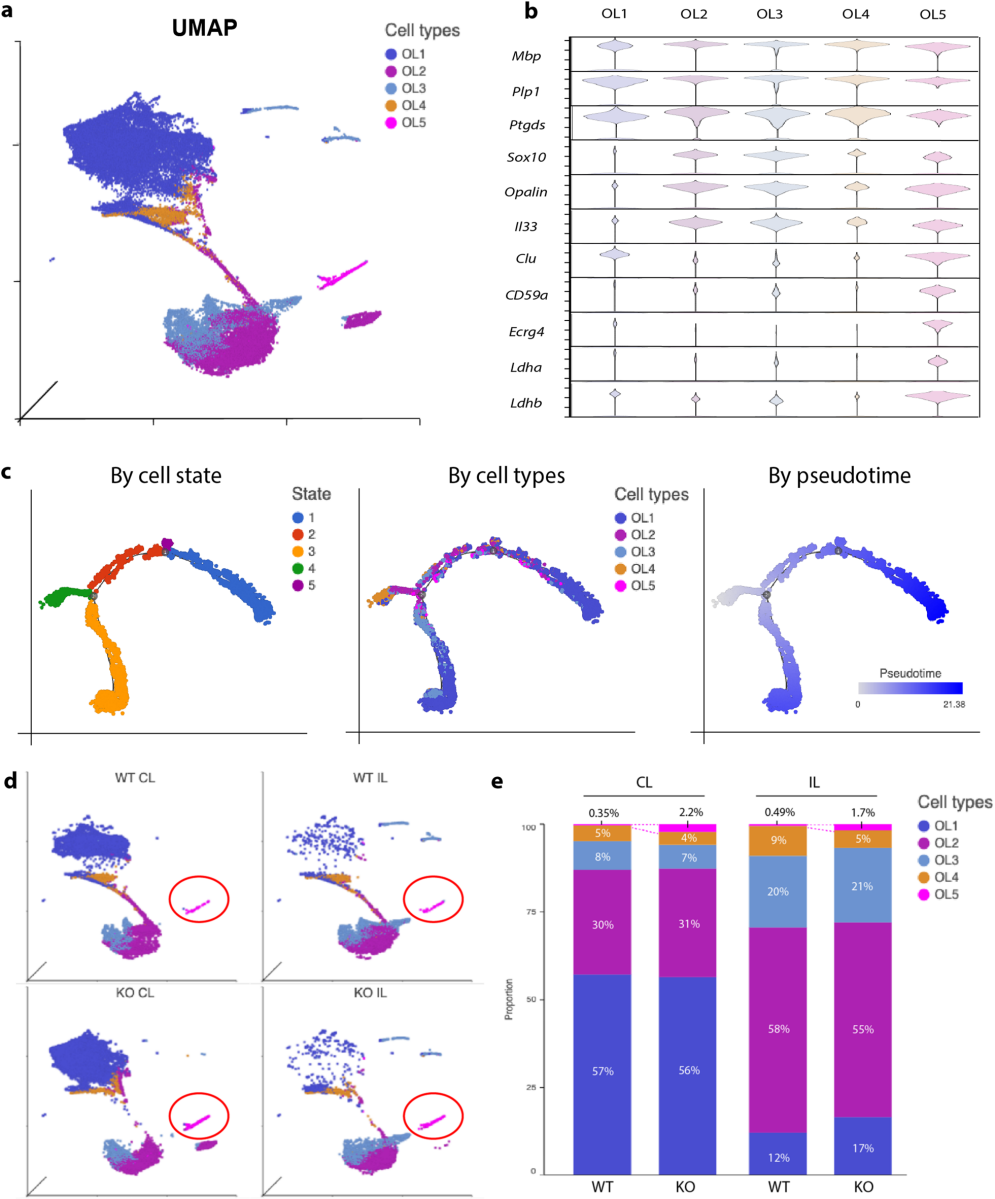
*Nhe1* cKO specifically expanded an oligodendrocyte subcluster with increased phagocytosis and lactate shuttling functions in post-stroke white matter tissues. **a** UMAP of oligodendrocyte subtypes from CL and IL hemispheres of WT and cKO white matter tissues. **b** Violin plots of signature genes for homeostatic oligodendrocytes and disease-associated oligodendrocytes (DAO) in each oligodendrocyte subtype. **c** Trajectory analysis of oligodendrocytes annotated by cell state, oligodendrocyte subtype, or developmental pseudotime. **d** Split view of UMAP of oligodendrocytes in CL or IL hemispheres of WT and cKO white matter tissues. **e** Proportion of oligodendrocyte subtypes in CL or IL hemispheres of WT and cKO white matter tissues

We previously observed enhanced white matter myelination in the *Nhe1* cKO brains after stroke [12, 13]. Here, we further studied white matter changes by dissecting the OL subclusters. All 5 transcriptionally distinct OL subclusters (OL1-5, Fig. 6a) expressed similarly high levels of *Mbp, Plp1, and Ptgds* (Fig. 6b), indicating a myelin-secreting phenotype [34]. OL1 likely represent the homeostatic mature OLs with concurrent low expressions of *Sox10, Opalin* and *Il33* (Fig. 6b) which typically indicate OPCs [35, 36]. Trajectory analysis with OL1 as the end state similarly illustrated 2 differential trajectories of OL development—starting from a common state of OL4 and through OL2, one arm matured into OL1 via OL3, while the other arm matured into OL1 via OL2 and OL5 as the transition states (Fig. 6c).

**Fig. 7:**
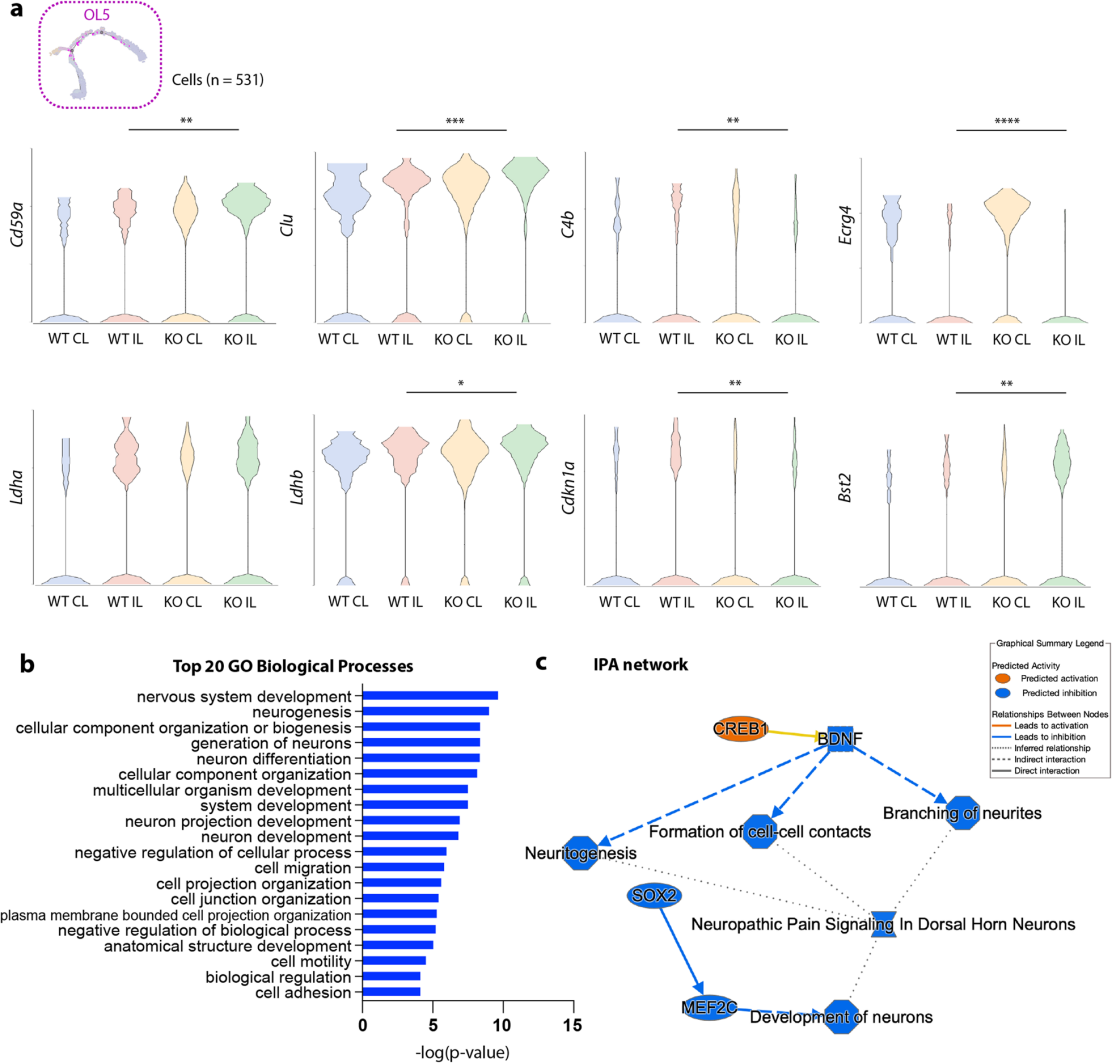
The OL5 subcluster specifically increased signature genes for disease-associated oligodendrocytes in the *Nhe1* cKO white matter tissues after stroke. **a** Violin plots showing *Nhe1* cKO displayed significantly elevated gene signatures for DAO within the OL5 subpopulation in post-stroke white matter tissues compared to WT. **b** GO analysis for the top 20 biological processes of DEGs from OL5 subpopulation. **c** IPA analysis of DEGs from OL5 subpopulation identified activated CREB1 signaling as the upstream regulator

Within the 5 OL subclusters, ischemic stroke significantly reduced the OL1 subgroup in both WT and cKO brains (Fig. 6d, e), possibly representing the acute loss (death) of mature OLs upon ischemic injury [37]. Concurrently, OL2 and OL3 subgroups were significantly increased compared to the non-stroke CL hemispheres (Fig. 6d, e), which may reflect the early recruitment and migration of OPCs to the injury site [37]. Interestingly, OL5 was the only OL subpopulation that showed a significant change between the WT and *Nhe1* cKO white matter tissues, which increased by 6.3-fold in the CL and 3.5-fold in the IL hemispheres (Fig. 6e). Compared to other OL subpopulations, OL5 was characterized by high expressions of a panel of genes involved in phagocytosis and lactate shuttling functions, such as *Clu, Cd59a, Ecrg4, Ldha, Ldhb,* etc. (Fig. 6b). This subcluster resembles the reported disease-associated oligodendrocyte (DAO) that have been characterized in neurodegenerative diseases, such as Alzheimer’s disease and multiple sclerosis [38–40]. Interestingly, *Nhe1* cKO white matter increased all these OL5-specific gene expressions, compared to those in the WT (Fig. 7a). GO analysis revealed neurogenesis and cell projection related functions as the most significantly altered biological processes (Fig. 7b, Additional file 1: Figure S5). IPA predicted activated CREB signaling to be a major upstream regulator (Fig. 7c), complementing our MG3 transcriptome analysis and flow cytometry study findings. Together, these data suggest that *Nhe1* cKO white matter specifically enhanced OL differentiation via the OL5 route, which encompasses phagocytosis and lactate shuttling functions that support white matter remyelination and axonal regeneration [41, 42].

### CellChat analysis revealed enhanced restorative microglia-oligodendrocyte crosstalk in *Nhe1* cKO white matter tissues

**Fig. 8:**
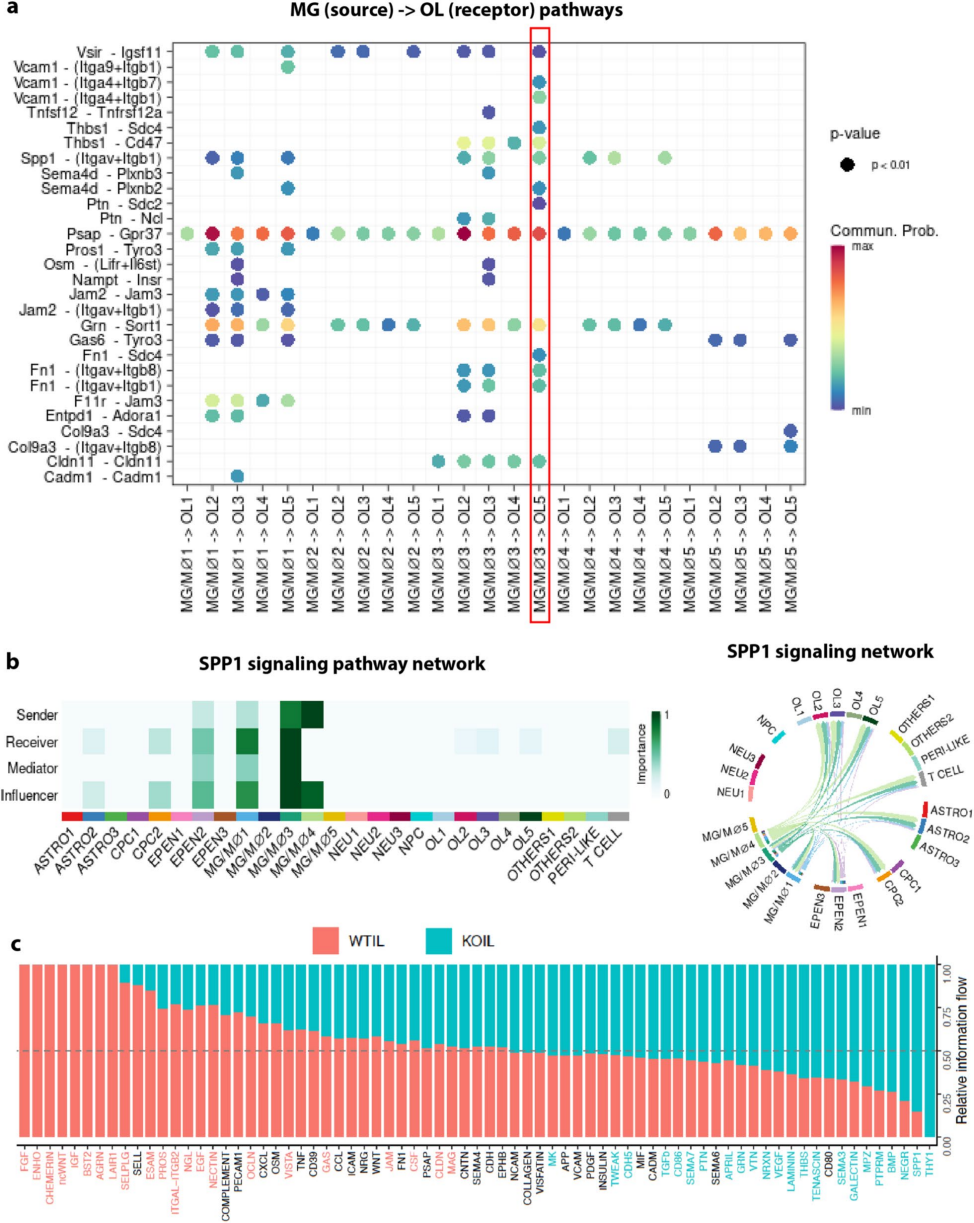
CellChat analysis revealed restorative microglia-oligodendrocyte crosstalk in the *Nhe1* cKO white matter tissues after stroke. **a** Heatmap revealed significant sender-receptor pathways from various microglial subclusters (MG1-5) to different oligodendrocyte subclusters (OL1-5). **b** Heatmap and chord diagram showing the involvement of different cell types and subclusters in the SPP1 signaling pathway network. **c** Relative information flow graph showing differential pathway regulations in the post-stroke white matter tissues of WT or *Nhe1* cKO brains, respectively

To better explore how the differential microglial subpopulations communicate with the different subgroups of oligodendrocytes in the post-stroke white matter tissues, we subjected our scRNAseq dataset to CellChat analysis, a well-established R toolkit to investigate ligand-receptor interactions to infer cell–cell communications [43]. Overall, the post-stroke *Nhe1* cKO white matter tissues had a higher number of interactions as well as interaction strength between different cell types (Additional file 1: Figure S6a, b). In particular, the interactions between MG3 and OL5, the two specifically elevated subpopulations in the *Nhe1* cKO white matter tissues, were highlighted (Fig. 8a, Additional file 1: Figure S6c). *Psap* (in microglia)—*Gpr37* (in oligodendrocytes) was the most outstanding source-receptor pathway that MG3 exerted on OL5 (Fig. 8a). Critically, *Psap* encodes the prosaposin protein, a multifunctional neuroprotective secreted protein and essential regulator for lysosomal trafficking and degradation functions in microglia [44, 45], aligning with our previously described observations of increased acidification (Fig. 4) and lysosomal function in *Nhe1* cKO microglia [13]. *Gpr37* is expressed in subsets of OPCs and newly formed immature OLs in adult mouse brain [46], while *Gpr37* KO mice exhibited reduced proliferation and differentiation of OPCs, and decreased number of OLs in the corpus callosum [46]. An enhanced interaction between microglial *Psap* and oligodendrocyte *Gpr37* may underlie the increased white matter remyelination we observed in the *Nhe1* cKO brains [12, 13]. Additionally, the CellChat analysis lends further support to our data in Figs. 2 and 3, showing MG3 as the most involved in SPP1 signaling pathway activation, compared to all the other cell types, with similarity to the homeostatic MG1 subpopulation (Fig. 8b). Relative information flow graph further demonstrated that SPP1 signaling pathway was the second most significantly upregulated pathways in the post-stroke *Nhe1* cKO white matter tissues compared to those in the WT (Fig. 8c). These data not only corroborated our findings that *Nhe1* cKO specifically expanded the MG3 subgroup associated with increased SPP1 pathways involved in myelin-supporting functions of microglia, but also unveiled possible novel mechanisms of the *Psap-Gpr37* ligand-receptor pairs in promoting white matter repair in post-stroke *Nhe1* cKO brains.

## Discussion

White matter tissues are defined by dense insultation of axonal fibers by the wrapping myelin-sheath secreted by mature oligodendrocytes. However, these white matter tissues are also maintained by other support cells residing within them, including astrocytes and microglial cells. Though microglia were traditionally assumed to be a homogenous cell population, next generation sequencing analyses have demonstrated a spectrum of transcriptional states with distinct subclusters emerging under different conditions, such as early brain development (termed “axon tract-associated microglia”, ATM) [20], Alzheimer’s disease pathogenesis (termed “disease-associated microglia, DAM) [17], and other neurodegenerative diseases or aging (termed “microglial neurodegenerative phenotype”, MGnD) [18]. scRNA-seq holds immense promise as a tool for investigating brain damage and recovery following ischemic stroke, not only by deepening our understanding of the complex genetic expression patterns and heterogeneity of cell responses, but also offering the potential to pave ways for future therapeutic developments with increased precision and less off-target complications. However, few studies had investigated brain cell heterogeneity in stroke conditions utilizing scRNA-seq [4, 5]. A recent scRNA-seq study in the ischemic hemispheres of adult male C57BL/6J mice successfully identified distinct principal cell clusters including microglia, oligodendrocytes, astrocytes, neutrophils, and CNS-associated macrophages, unveiling their respective cell type subpopulations and differential gene expression patterns within 24 h after tMCAO [5]. In another stroke scRNA-seq study, the authors dissected immune cell responses in the brain border compartments compared to the brain parenchyma and discovered a unique phenotype of myeloid cells involved in the inflammatory response to injury, termed “stroke-associated myeloid cells” (SAMC) [47], which also presented internalized myelin droplets in the brains of human stroke patients. Despite these efforts, most of the studies were conducted in hemispheric brain tissues, and there is a lack of scRNAseq analysis specially in white matter tissues, where cellular compositions and responses to stroke injury can vary significantly compared to those in the gray matter.

In this study, we identified a novel population of microglia (MG3) that only appeared in the white matter tissues in response to stroke (in both WT and cKO) but not in the non-stroke white matter tissues (Fig. 3c). These stroke-associated white matter microglia (SAWM) showed lower expressions of the homeostatic microglial markers (*P2ry12, Tmem119,* etc.) along with elevated expressions of the DAM- and/or MGnD-related genes (*Apoe, Trem2, Ctsb, Ctsd*, etc.) [17] (Fig. 2b). Most interestingly, compared to other microglial subclusters, these SAWM also exclusively increased all the signature genes for ATM [20], highlighted by the high expressions of *Spp1, Gpnmb, Lgals3, and Lgals1* (Fig. 3b), where the ATM subcluster of microglia was reported to only appear during a restricted developmental window before myelination occurred, while the tissues in which they were concentrated eventually became heavily myelinated, such as the corpus callosum and cerebellum [20]. In addition, among all cell types and/or subclusters, the SAWM population was the most highly involved in the SPP1 signaling pathway network, similar to those in the homeostatic MG1 subcluster (Fig. 8b), further validating the similarity to the physiological microglial phenotype in supporting white matter remyelination. In summary, here we not only revealed a complete landscape of the cell heterogeneity specifically in the white matter tissues after stroke, but also identified the novel SAWM population that could be critical to exert white matter repairing functions after stroke.

Our prior work found that the *Nhe1* gene plays a critical role in the fine-tuning of microglial profiles and that NHE1 protein inhibition promotes white matter remyelination after ischemic stroke or traumatic brain injury [12, 13, 48]. Here we further investigated the mechanisms underlying these SAWM population transformation by deleting the *Nhe1* gene selectively in microglia. NHE1 protein mediates the electroneutral transport of H^+^ efflux in exchange for Na^+^ influx and is one of the major pH_i_ regulators in microglia [49]. We identified changes to microglial acid–base homeostasis and transcriptomic profiles in the generic CD11b^+^CD45^+^ microglial population as well as the SAWM-specific CD11c^+^ subpopulation in *Nhe1* cKO brains, including increased acidification and upregulated CREB signaling with elevated metabolism in these cells. Regarding how pH_i_ changes affect CREB signaling activation, studies characterizing the metabolic derangements in cancer cells have shown that decreased pH_i_ (as well as increased extracellular pH) promotes CREB signaling activation which in turn drives cellular metabolism across models of brain injury [21, 50]. Our group has detected elevated oxidative phosphorylation energy metabolism in the *Nhe1* cKO microglia after stroke [13]. Concurrently, we found significantly increased CREB1 activation/phosphorylation in the *Nhe1* cKO CD11b^+^CD45^int^ cells compared to WT control. Moreover, a significantly higher proportion of p-CREB1-expressing CD11b^+^CD45^int^ also increased BDNF expression, a growth factor proven to be essential for white matter integrity in human patients [51, 52]. Although depletion of microglial BDNF and BDNF supplementation experiments generated different outcomes in different disease models, BDNF (and the upstream Akt/CREB signaling) appears to be a critical player in microglial activation and proliferation in response to stress/injury [53–55]. Other possible mechanisms underlying CREB1 activation include reports that NHE1 protein activation can generate a periodic intracellular Ca^2+^ increase (Ca^2+^ oscillation), leading to Ca^2+^/CaMKII-dependent CREB activation in brain pericytes [56]. However, we previously reported that NHE1 blockade with either its potent inhibitor HOE642 [49, 57] or global NHE1 knockout [58] almost completely attenuated the Ca^2+^ increase in cultured neurons, microglia, or astrocytes after in vitro ischemia, likely through blocking the reversed operation of Na^+^/Ca^2+^ exchanger (NCX_rev_), which shall mitigate the Ca^2+^/CaMKII-dependent CREB activation. Further investigation is needed to depict the specific mechanisms underlying the CREB1 activation in *Nhe1* cKO microglia/macrophages.

Recent studies have reported a specialized group of oligodendrocytes, termed “disease-associated oligodendrocyte” (DAO), that participate in phagocytosis and lactate shuttling functions which support white matter remyelination and axonal regeneration [41, 42] in the context of neurodegenerative diseases, such as Alzheimer’s disease and multiple sclerosis [38–40]. We found that a subgroup of oligodendrocytes (OL5) exhibited increased expressions of these DAO-associated genes such as *Clu, Cd59a, Ecrg4, Ldha, Ldhb,* etc. [38–40] in both stroke and non-stroke hemispheres. Most interestingly, pathway analysis by IPA for the OL5 subpopulation predicted activated CREB1 signaling to be the most significant upstream regulator, which corroborated our microglial findings. In summary, our data suggest that selective deletion of microglial *Nhe1* could exert its effects on white matter myelination by stimulating microglial CREB1 pathway activation and BDNF secretion, which accelerated restorative microglia-oligodendrocyte communications.

In our study, it is worth noting that the trends of CREB1 signaling activation persisted to be significantly increased in both CD11b^+^CD45^int^ microglia and CD11b^+^CD45^hi^ microglia/macrophage populations in the post-stroke cKO brains. The pHrodo experiments revealed statistically significant acidification in the combined CD11b^+^CD45^+^ population, as well as in the CD11c^+^ microglial populations (p < 0.05) in the cKO brains (CL and IL hemispheres), compared to the WT brains. But the changes in pH_i_ did not reach significance when CD11b^+^CD45^int^ and CD11b^+^CD45^hi^ microglia/macrophages in WT and cKO brains were separately analyzed. This could be due to large variability in the data with relative low n values. Nevertheless, as no differences were detected in pH_i_ in either CD11b^+^/CD45^+^ or CD11c^+^ populations between sham-operated WT or cKO brains (Additional file 1: Figure S4a), the increased pHrodo fluorescent intensity (indicating acidified pH_i_) in both CL and IL hemisphere of cKO brains are stroke-dependent. This is consistent with previous report that the non-lesioned CL hemispheres showed robust defect in electrophysiology recordings in human stroke patients, which correlate with poorer outcomes [59]. In comparison, less acidification in the CD11c^+^ microglia was detected in the non-stroke CL hemisphere of *Nhe1* cKO brains, where stroke was able to induce further acidification (Fig. 4b, c). Thus, we cannot rule out that additional mechanisms may be involved in regulating pH in the CD11c^+^ microglia. It is reported that Hv1 only mediated large proton currents in microglia, but not in neurons or astrocytes, indicating a relative selectivity of Hv1 expression in microglia [60]. Global knockout of Hv1 reduced brain damages and conferred neuroprotection after ischemic stroke [60], spinal cord injury [61], or traumatic brain injury [62] via similar mechanisms of preventing oxidative damage [63]. While *Hvcn1* (encoding Hv1) gene expression in MG3 subpopulation is relatively low compared to other microglia/macrophages (MG1, 2, 4, and 5; 2.13-fold decrease, p < 0.0001), we found a small but significant elevation of *Hvcn1* gene expression in the MG3 of cKO brains post-stroke (1.32-fold increase, p < 0.01; Additional file 1: Figure S7), which shall alkalinize pH_i_. Other potential pH_i_ regulating mechanism includes the Na^+^/HCO3^−^ cotransporter (NBCe1), which facilitates HCO3^−^ outward transport (coupled with Na^+^ efflux) to buffer extracellular H^+^ loads in response to neuronal activity [64], or stimulates inwardly directed HCO3^−^ transport (coupled with Na^+^ influx) in ischemic stroke conditions [65]. We detected similarly low levels of *Slc4a4* (encoding NBCe1) gene expression in MG3 compared to other microglia/macrophage subpopulations, but stroke triggered a small but significant increase of *Slc4a4* gene expression in the MG3 of cKO brains (1.36-fold increase, p < 0.01; Additional file 1: Figure S7). Whether the elevated gene expression translates to a higher NBCe1 protein expression, and whether the NBCe1 activity is in outward mode (in compensation of NHE1 blockade) or inward mode (triggered by stroke) remain unknown and warrant further exploration. Overall, our new findings support a novel mechanism of pH regulation in promoting restorative microglial transformation and growth factor release, shedding light on pH regulation as a novel therapeutic approach in stroke treatment.

There are several limitations in this study. First, our overview UMAP reveals the presence of neurons in the dissected tissues. During our tissue dissection process, white matter tracts (CC and EC) were visually identified and manually dissected from brain sections at 4 different bregma levels (Additional file 1). Therefore, there are inevitably some contamination of non-white matter tissues (cortex and/or hippocampal tissues) in the single cell suspension. We focused our analysis on the MG and OL populations, where neuronal contamination imposed minimum effects. Secondly, astrocytes are the most abundant glial cell type in the human brains, however the transcriptome changes of astrocytes and their roles in white matter damage/repair are not fully explored here. Astrocytes can contribute to white matter damage through either direct cell–cell communication with oligodendrocytes, or by indirectly affecting microgliosis through protein accumulation, unbalanced secretion of a variety of molecules, including extracellular matrix proteins, pro- and anti-inflammatory cytokines and chemokines [66]. Astrocyte may also alter the gap junctional network and change ionic and nutrient homeostasis [66]. Therefore, the astrocyte-oligodendrocyte interactions warrant further investigation in future studies. Lastly, despite the significant improvements in white matter myelination in the post-stroke *Nhe1* cKO brains [12, 13], the only significantly altered subgroup of oligodendrocytes in the *Nhe1* cKO white matter tissues compared to the WT white matter, was OL5. While we saw several folds of expansion of the OL5 subcluster in the *Nhe1* cKO white matter (6.3-fold in the CL and 3.5-fold in the IL hemispheres), the cell numbers of the OL5 subgroup were relatively small, only consisting of less than 3% of total oligodendrocytes. However, this is consistent with other report showing that the distinct DAO population does not exceed 5% of all oligodendrocytes [67], which is also consistently shared across multiple pathologies, including different models of Alzheimer’s disease (5xFAD amyloidosis model, P301L pure tauopathy model, and PS2/APP/P301L combined tauopathy/amyloidosis model), a multiple sclerosis model of experimental autoimmune encephalomyelitis, or following LPS-induced brain inflammation [67], with signature genes involved in phagocytosis and lactate shuttling functions [38–40]. Further in-depth studies are required to determine the roles and mechanisms of this small OL population in relation to disease progression/repair. In addition, the genetic profiles within each OL subgroups may not be homogenous between the WT and cKO groups, despite their similar cell numbers. Thus, further analyses on the other OL subgroups (OL1-4) are also warranted. Overall, our scRNAseq transcriptome study revealed new possible cellular mechanisms between microglia-oligodendrocyte interactions, which will be further validated using microglia-oligodendrocyte in vitro co-culture system in future studies.

## Conclusion

**Fig. 9:**
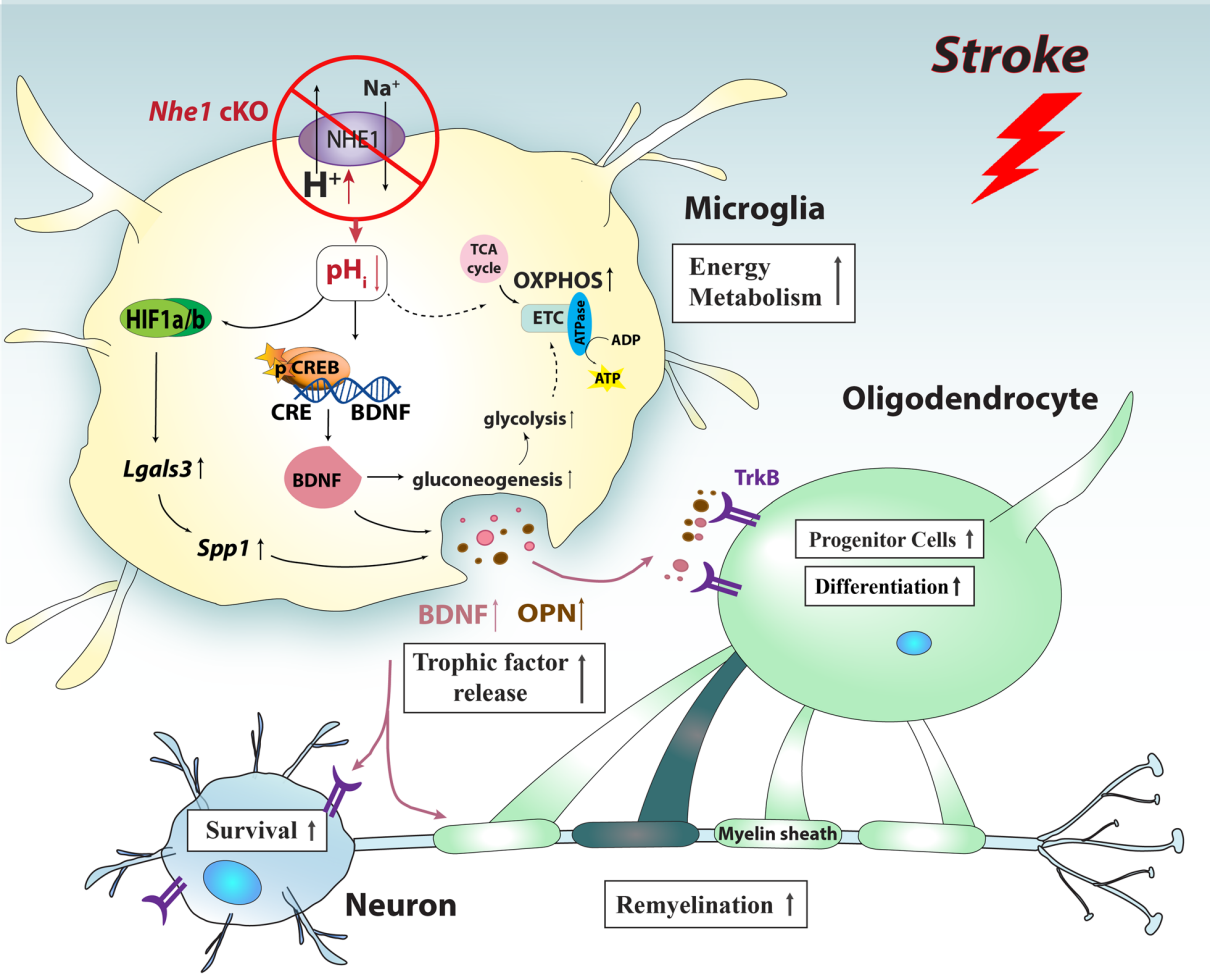
Schematic illustration of NHE-1-mediated pH_i_ and signaling regulation in microglial cells and communication with oligodendrocytes in ischemic white matter repair. Using single cell RNAseq, we discovered that selective deletion of microglial *Nhe1* acidified microglia and activated CREB signaling with increased BDNF secretion, and elevated SPP1 signaling pathway, which enhanced the restorative microglia-oligodendrocyte interactions in support of white matter remyelination. Our findings identified a novel group of stroke-associated white matter microglia, which can be activated by acidifying microglial pH_i_ through blocking the NHE1 protein. Regulating microglial pH_i_ presents a possible mechanism in promoting the beneficial microglia-oligodendrocyte interactions in improving white matter myelination after ischemic stroke

In this study, taking advantage of the advanced scRNAseq technology, we were able to dissect subclusters of microglia and their differential roles in communication with oligodendrocytes in the post-stroke white matter tissues. Specifically, we found selective deletion of *Nhe1* from *Cx3cr1*^+^ cells acidified microglia/macrophage and increased CREB signaling in a specific subgroup of microglia with elevated expression of myelin-supporting functions as well as lipid metabolism, phagocytosis, and lysosomal genes. Concurrently, we detected stimulated OL subgroup with increased phagocytosis and lactate shuttling functions in white matter tissues of *Nhe1* cKO mice, and elevated SPP1 signaling pathway for microglia-oligodendrocyte communication revealed by CellChat analysis. Taken together, our findings demonstrated that blocking the NHE1 protein can acidify pH_i_ in a specific group of stroke-associated microglia/macrophage. Regulating microglial pH_i_ presents a possible mechanism in promoting the beneficial microglia-oligodendrocyte interactions in improving white matter myelination after ischemic stroke (Fig. 9).

## Material and methods

### Animals

All animal experiments and procedures were approved by the University of Pittsburgh Institutional Animal Care and Use Committee and performed in accordance with the National Institutes of Health Guide for the Care and Use of Laboratory Animals. Tamoxifen-injected *Cx3cr1-Cre*^*ER*±^*;Nhe1*^*flox/flox*^ mice and *Cx3cr1-Cre*^*ER*±^ mice were used as *Nhe1* cKO and WT control groups, respectively, as described in Additional file 1.

### Transient focal ischemia model

Transient focal ischemic stroke in mice was induced by 60-min transient middle cerebral artery occlusion (tMCAO) as described in Additional file 1.

### Single cell RNA-sequencing and bioinformatic analyses

Single cell RNA-sequencing (scRNAseq) and bioinformatic analyses of white matter tissues from WT and *Nhe1* cKO mouse brains at 3 days post-tMCAO were performed with droplet-based scRNAseq (10 × Genomics), as described in Additional file 1. Briefly, the quality of the single-cell suspension was confirmed prior to library preparation using a Chromium Next GEM Single Cell 3ʹ Reagent kit v3.1. The samples were then sequenced on an Illumina Novaseq 6000 PE150 (Read 1 150 bp, i7 Index 10 bp, i5 Index 10 bp, Read 2 150 bp). Cell Ranger (10 × Genomics) analysis pipeline was used to perform alignment, filtering and counting of barcodes, and unique molecular identifiers. The sequencing data was aligned to the mouse genome mm10. Count matrices generated by Cell Ranger was imported into Partek Flow 8.0 for further analysis. DESeq2 was performed to identify differentially expressed genes (DEGs) with a criteria of FDR p < 0.05 and fold change > 2 or < -2. Monocle2 was conducted for trajectory analysis. Gene Ontology (GO) analysis, Gene Set Enrichment Analysis (GSEA), and Ingenuity Pathway Analysis (IPA, Qiagen Bioinformatics, Germany) were conducted as described in Additional file 1.

For cell–cell communication analysis and visualization, combined counts from all post-stroke samples (both WT and cKO) were exported and analyzed using the CellChat package (v1.5.0, https://github.com/sqjin/CellChat, accessed July 2023) in R. Briefly, unique CellChat objects were created encompassing cells in the post-stroke hemispheres of WT and cKO groups, respectively, along with one object combining cells from both datasets. Detailed methods are described in Additional file 1. The sequencing data have been deposited to the Gene Expression Omnibus (GEO) database with experiment series accession number GSE247102.

### Flow cytometry

Microglial intracellular pH (pH_i_) was measured with a novel pH indicator, pHrodo-Red (Thermo Fisher Scientific, USA) at 3 days post-stroke using flow cytometry. Briefly, single cell suspensions were stained with CD11b-APC (Invitrogen, USA), CD45-PerCP-Cy5.5 (BioLegend, USA), and CD11c-BV510 (BioLegend, USA) antibodies for 20 min at 4 °C, and subsequently incubated with pHrodo-Red for 30 min at 37 °C. Median fluorescent intensity (MFI) of pHrodo within the CD11b^+^/CD45^+^ or CD11c^+^ microglia/macrophages populations were recorded in an LSR Fortessa flow cytometer.

For validation of the CREB signaling pathway at a protein level, single cell suspensions were stained with CD11b-BV421 (BioLegend, USA), CD45-PerCP-Cy5.5 (BioLegend, USA), and CREB1-FITC antibodies for 20 min at 4 °C. The cells suspensions were permeabilized and fixed before an intracellular staining was conducted with a rabbit anti-mouse BDNF antibody (Invitrogen, USA) before incubating with a donkey anti-rabbit IgG-PE (BioLegend, USA) antibody for 20 min at 4 °C. Detail methods are described in Additional file 1.

### Data analysis

Unbiased study design and analyses were used in all the experiments. Blinding of investigators to experimental groups were maintained until data were fully analyzed whenever possible. Data were expressed as mean ± SEM (GraphPad Prism, USA). Two-way ANOVA analysis was used, and a p value < 0.05 was considered statistically significant. All data were included unless appropriate outlier analysis suggested otherwise.

### Supplementary Information

**Additional file 1.** Supplementary Information.

## Data Availability

Sequencing data have been deposited to the Gene Expression Omnibus (GEO) database with experiment series accession number GSE247102. All data needed to evaluate the conclusions in the paper are present in the paper and/or additional materials.
