# Supplementary material for: Deletion of Slc9a1 in Cx3cr1+ cells stimulated microglial subcluster CREB1 signaling and microglia-oligodendrocyte crosstalk
Source: J Neuroinflammation. 2024 Mar 20;21:69. doi: 10.1186/s12974-024-03065-z (PMC10953158; doi:10.1186/s12974-024-03065-z)
Supplement: Supplementary file 1 — Additional file 1. Supplementary Information. [file 12974_2024_3065_MOESM1_ESM.docx]

**Additional Materials and Methods**

***Animals***

All animal studies were approved by the University of Pittsburgh Medical Center Institutional Animal Care and Use Committee, which adheres to the National Institutes of Health Guide for the Care and Use of Laboratory Animals, and reported in accordance with the Animal Research: Reporting In Vivo Experiments (ARRIVE) guidelines ^1^. Animals were provided with food and water ad libitum and maintained in a temperature-controlled environment in a 12/12 h light-dark cycle. All efforts were made to minimize animal suffering and the number of animals used.

*Cx3cr1-CreER^+/-^* (wild-type, WT) control mice and *Cx3cr1-CreER^+/-^;Nhe1^f/f^* (*Nhe1* cKO) mice were established as described previously ^2^. Both genotypes of mice (male or female) at postnatal day 30-40 (P30-40) received tamoxifen (Tam, Sigma) (75 mg/kg body weight/day at a concentration of 20 mg/ml in corn oil, intraperitoneally) for 5 consecutive days. *Cx3cr1* is expressed by both brain resident microglia and peripheral infiltrating bone marrow-derived myeloid (BMDM) cells. However, BMDM cells, but not the *Cx3cr1^+^* microglia, have a 21-day self-renewing cycle^7^. Therefore, in this study, we implemented a 30-day post-injection waiting period was given for clearance of Tam ^3-5^ and for replenishing of the *Cx3cr1^+^* monocytes ^6^ prior to induction of ischemic stroke (**Fig. 1A**). This method has been proven to achieve a complete replenishment of the *Cx3cr1^+^* BMDM ^6^ and is effective in our study ^2,8^ as well as in others ^9-11^. Surgeries were performed by investigators blinded to mouse genotype and experimental group assignments.

***Transient focal ischemia model***

Focal cerebral ischemia was induced by occlusion of the left middle cerebral artery (MCA) as described before ^2,12^. Briefly, mice were kept under 1.5% isoflurane anesthesia during the procedure and the core temperature (37.0 °C) was maintained by a small animal temperature controller pad throughout all procedures. After the midline skin incision, the left common carotid artery was exposed and the superior thyroid artery and occipital artery branches of the external carotid artery were isolated and coagulated. The animals were subjected to MCA occlusion (MCAO) by the introduction of a silicone-coated suture (6-0 monofilament nylon, Doccol, USA) inserted via the external carotid artery. Reperfusion was established by the withdrawal of the filament after 60 min of transient MCAO (tMCAO). The incision was closed and the mice were allowed to recover under a heating lamp to maintain the core temperature (36~37 °C) during a 30-60 min recovery period. All animals were returned to their cages with free access to food and water after the procedures.

***Single-cell RNA sequencing and bioinformatics analyses***

Single-cell RNA sequencing and bioinformatic analysis were performed in white matter tissues dissected from the WT or *Nhe1* cKO brains at 3 days post-tMCAO. Briefly, mice were euthanized with an overdose of CO_2_ and transcardially perfused with ice-cold saline, as described before ^2^. The brains were dissected and cut into 4 coronal slices of 2 mm thickness in a brain slicer matrix. Cortices were removed and white matter tissues (corpus callosum and external capsules) were separated from the contralateral and ipsilateral hemispheres and dissociated into single-cell suspensions using a Neural Tissue Dissociation Kit with the gentleMAC Octo Dissociator (Miltenyi Biotech Inc., Germany). Myelin was removed using the 30/70 Percoll gradient method as described ^2^. Single-cell suspension was prepared as we described before ^13^ and used for droplet-based scRNAseq (10x Genomics). The quality of the single-cell suspension was confirmed prior to the start of library preparation with an acridine-orange-propidium iodide stain (Nexcelcom Biosciences) and a Cellometer Auto 2000 Viability Counter (Nexcelcom Biosciences). Library Preparation (Chromium Next GEM Single Cell 3’ Reagent kit v3.1) was conducted as per the manufacturer’s protocol. The quality of the cDNA libraries was verified using a High Sensitivity D5000 ScreenTape and 4200 Tapestation system (Agilent Technologies). Samples were sequenced on an Illumina Novaseq 6000 PE150 (Read 1 150bp, i7 Index 10bp, i5 Index 10bp, Read 2 150bp). Cell Ranger (10x Genomics) analysis pipeline was used to perform alignment, filtering and counting of barcodes, and unique molecular identifiers. The sequencing data was aligned to the mouse genome mm10. Count matrices generated by Cell Ranger were imported into Partek Flow 8.0 for further analysis.

Further QC was conducted in Partek Flow to remove low-quality cells, filtered by unique molecular identifiers (UMI), detected features, and mitochondrial counts. After normalization, principal component analysis (PCA) was conducted and Harmony was used to remove batch effects. DESeq2 was performed to identify differentially expressed genes (DEGs) with criteria of FDR p < 0.05 and fold change > 2 or < -2. Monocle2 was conducted for trajectory analysis. Gene Ontology (GO) analysis, Gene Set Enrichment Analysis (GSEA), and Ingenuity Pathway Analysis (IPA, Qiagen Bioinformatics, Germany) were conducted as described.

For cell-cell communication analysis and visualization, counts from the ipsilateral hemispheres were combined separately for samples in the WT and cKO groups for analysis using the CellChat package (v1.5.0, <https://github.com/sqjin/CellChat>, accessed July 2023) in R. We used CellChat’s native database for mice to create two CellChat objects encompassing cells in the WT and cKO groups respectively, as well as one object combining datasets for both genotypes. In order to determine whether the total number of cell interactions were altered between different genotypes, we use default parameters to compare the number and strengths of interactions using the compareInteractions function. We also determined and visualized the differential number of interactions and their strength in the cell-cell communication network between the WT and cKO groups using netVisual_diffInteraction. These interactions were mapped by source/sender cell groups with the netVisual_heatmap function. Using the rankNet function we determined the conserved pathways between groups and visualized the extent they contribute to information in each genotype. The combined dataset was analyzed according to the workflow established by Jin et al ^14^ to show all significant ligand-receptor interactions from the microglial subclusters (MG/MØ) to the oligodendrocyte subclusters (OL) as well as the cell groups involved in the SPP1 pathway, which was visualized as the computed centrality scores. The sequencing data have been deposited to the Gene Expression Omnibus (GEO) database with experiment series accession number GSE247102.

***Flow cytometry***

Mice were euthanized with an overdose of CO_2_ and transcardially perfused with ice-cold saline at 3 days post-stroke, as described before ^2^. After the removal of cerebellum and meninges, CL and IL hemispheric tissues were separated and dissociated into single-cell suspensions using a neural tissue dissociation kit with the gentleMAC Octo Dissociator (Miltenyi Biotech Inc., Germany). Myelin was removed using the 30/70 Percoll gradient method as described ^2^. Microglial intracellular pH (pH_i_) was measured with a novel pH indicator, pHrodo-Red (Thermo Fisher Scientific, USA). Briefly, single-cell suspensions were stained with CD11b-APC (Invitrogen, USA), CD45-PerCP-Cy5.5 (BioLegend, USA), and CD11c-BV510 (BioLegend, USA) antibodies for 20 min at 4 ^o^C, and subsequently incubated with pHrodo-Red for 30 min at 37 ^o^C. For validation of the CREB signaling pathway at a protein level, single cell suspensions were stained with CD11b-BV421 (BioLegend, USA), CD45-PerCP-Cy5.5 (BioLegend, USA), and CREB1-FITC antibodies for 20 min at 4 ^o^C. The cell suspensions were permeabilized and fixed before intracellular staining was conducted with a rabbit anti-mouse BDNF antibody (Invitrogen, USA) before incubating with a donkey anti-rabbit IgG-PE (BioLegend, USA) antibody for 20 min at 4 ^o^C. Median fluorescent intensity (MFI) of pHrodo and CREB1/BDNF expressions within the CD11b^+^/CD45^+^ or CD11c^+^ microglia/macrophages populations were recorded in an LSRII flow cytometer (BD Biosciences, USA) running FACS Diva software (BD Biosciences, USA) with the following settings: Forward scatter (FSC) V = 425, mode = Lin; Side scatter (SSC) V = 225, mode = Lin; FITC V = 525, mode = Log; BV421 V = 375, mode = Log; PerCP-Cy5.5 V = 570, mode = Log; APC V = 575, mode = Log; PE V = 600, mode = Log. In each experiment, at least 100,000 events were recorded from each sample for analysis. Data were then analyzed in FlowJo software (v10.4.0, BD Biosciences, USA). All data were analyzed after live cells and single cells gating as described before ^15,16^.

**
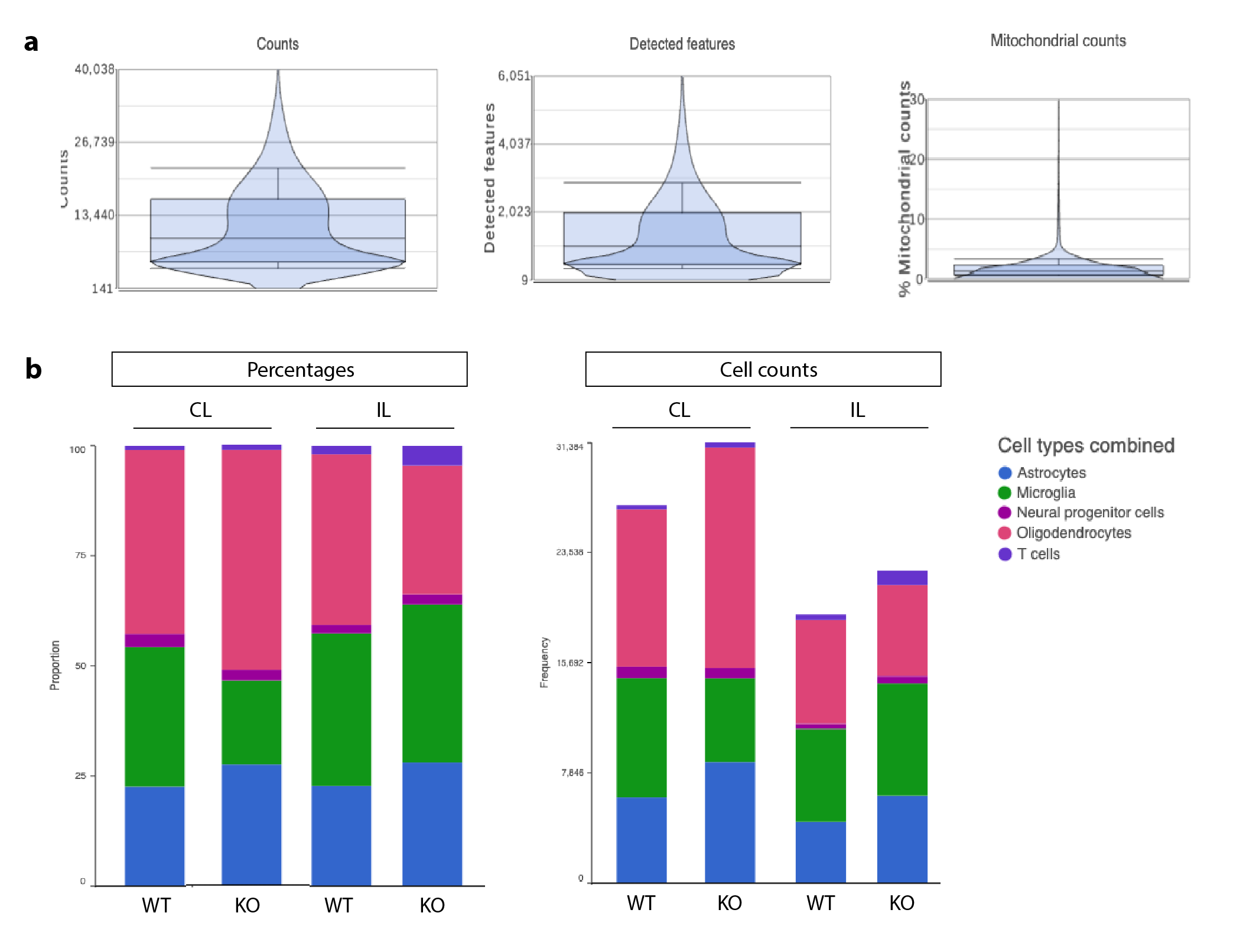
**

**Fig. S1. scRNAseq identified various cell types in post-stroke white matter tissues.**

**a.** Unique molecular identifiers (UMI) counts, detected features, and mitochondrial percentages after quality control. **b.** Proportion and absolute cell counts of each combined cell type after removing potential contaminating cell types in contralateral (CL) and ipsilateral (IL) white matter tissues of WT and cKO stroke mice at 3 d post-tMCAO.

**
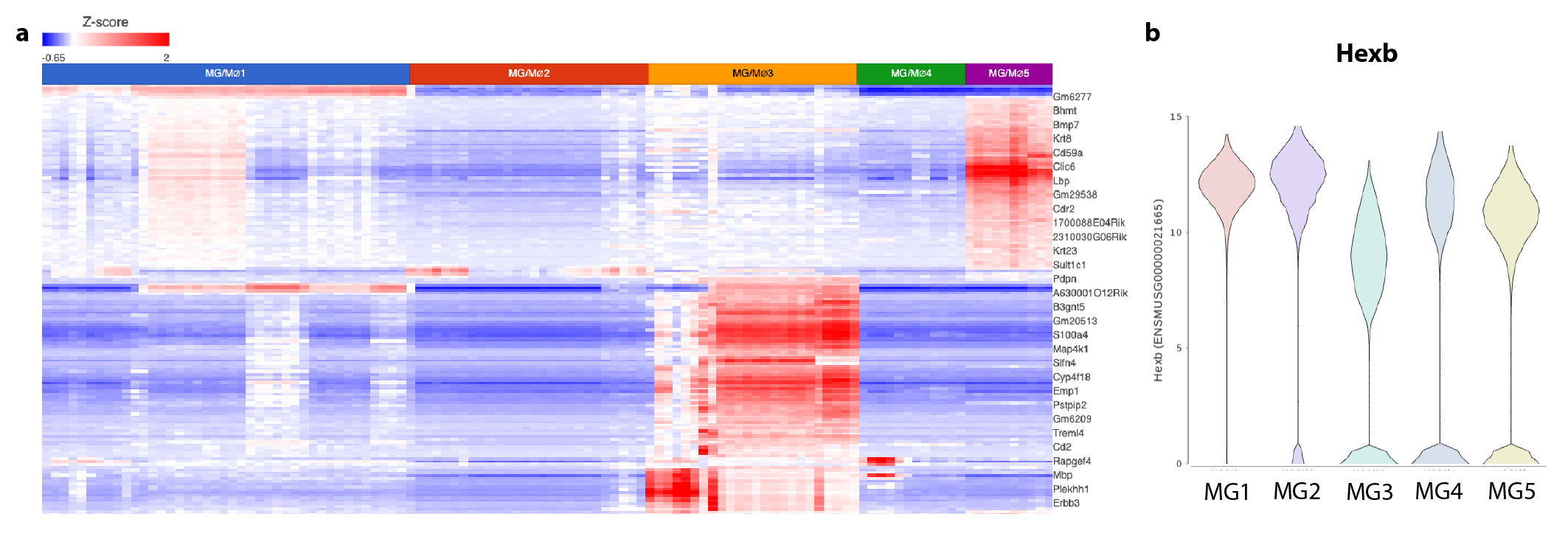
**

**Fig. S2. scRNAseq identified transcriptionally distinct microglial subclusters.**

**a.** scRNAseq identified five transcriptionally distinct microglial subclusters. **b.** Consistently high expression of the microglial specific marker *Hexb* in all five microglial subclusters. Data are from the same dataset as **Figure 1**.

**
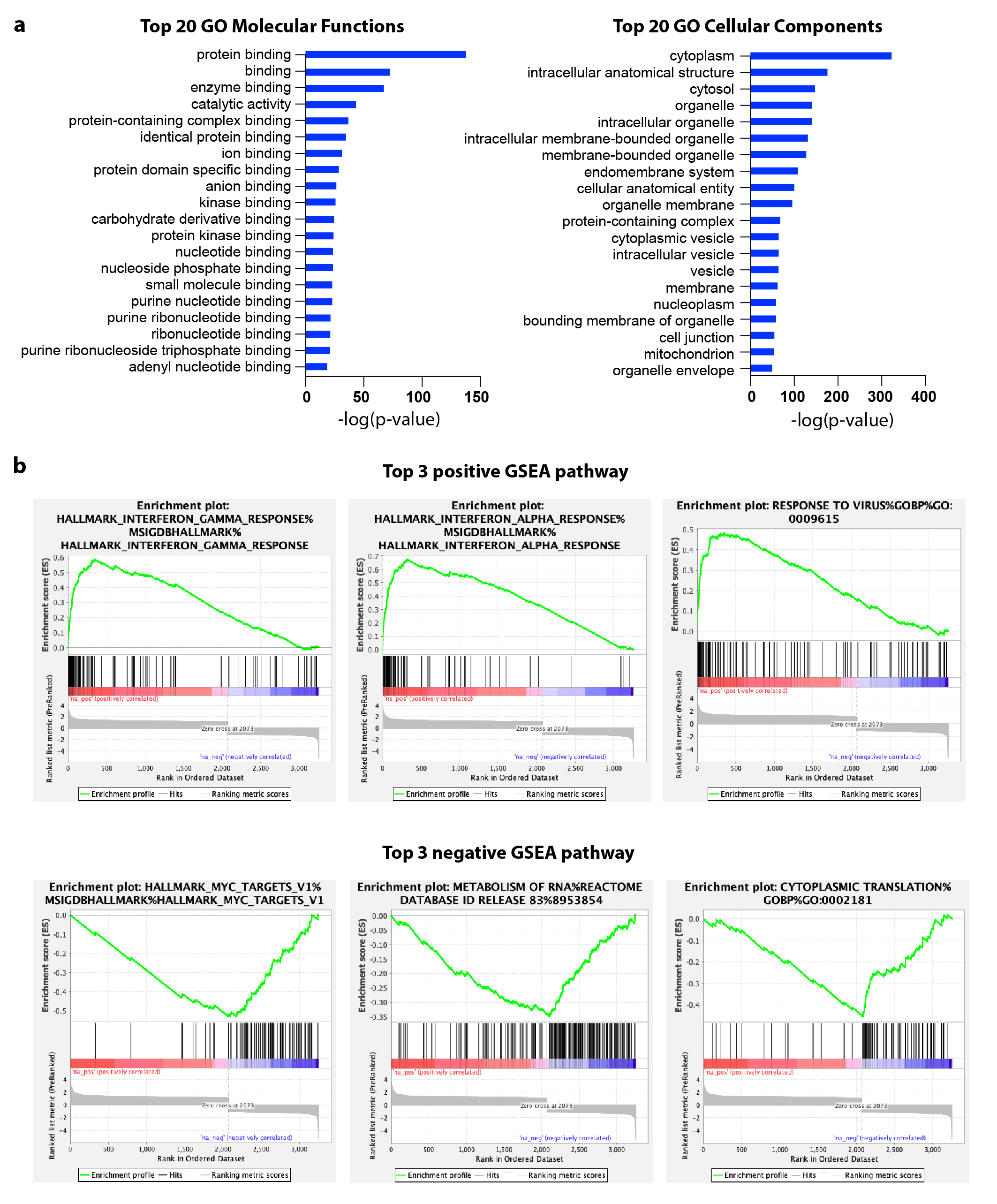
**

**Fig. S3. Transcriptomic pathway analysis with DEGs of MG3 subpopulation.**

**a.** Top 20 molecular functions and cellular components in the Gene Ontology (GO) analysis of MG3 subpopulation from post-stroke white matter tissues (WT and cKO) at 3 d post-tMCAO. **b.** Top 3 positively and negatively regulated pathways by Gene Set Enrichment Analysis (GSEA). Data are from the same dataset as **Figure 1.**

**
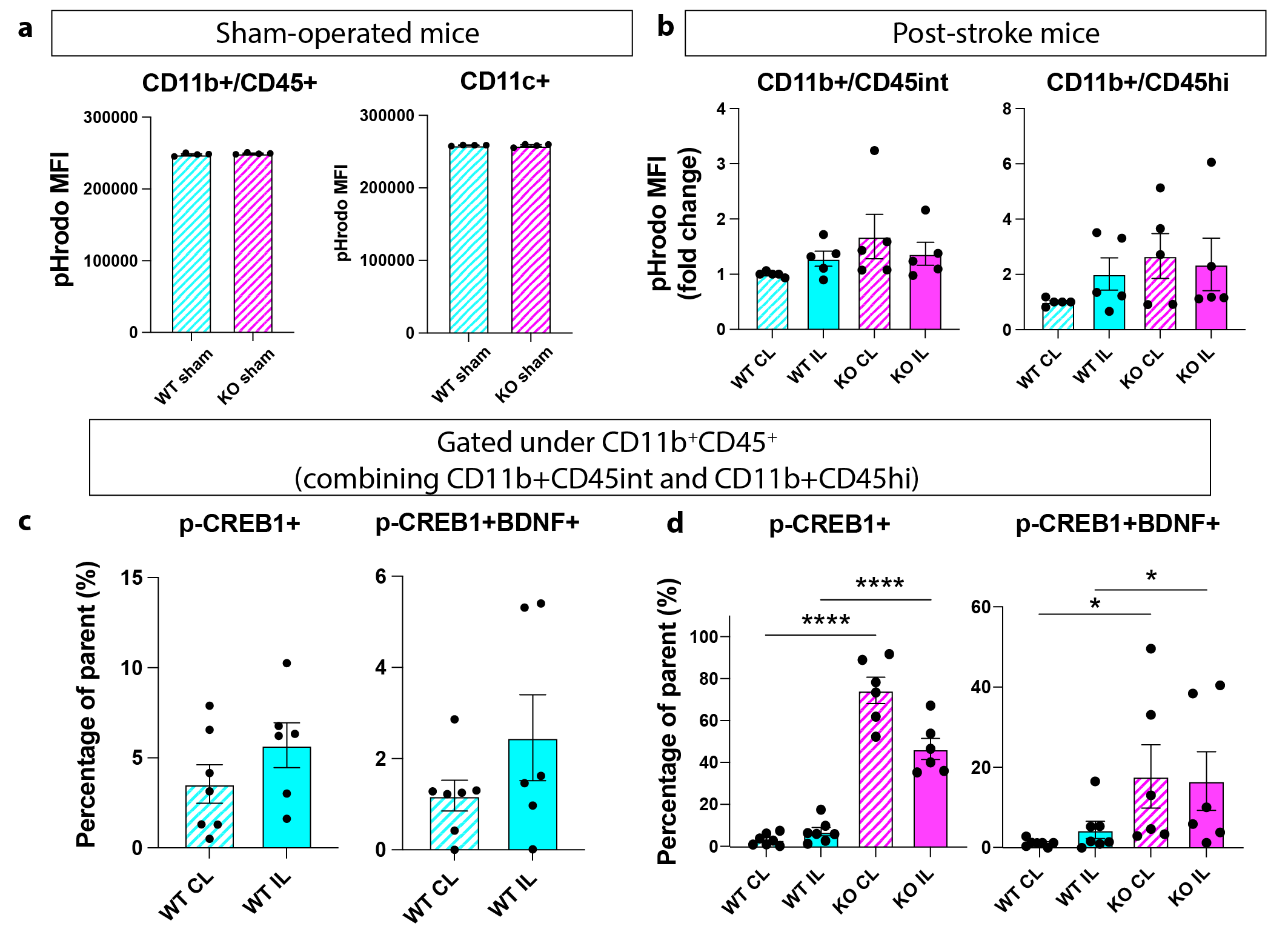
**

**Fig. S4. Additional analysis of pH_i_ and CREB1/BDNF pathway expressions in microglia/macrophages at 3 days post-stroke.**

**a.** Mean fluorescent intensity (MFI) of pHrodo^+^ cells within CD11b^+^CD45^+^ or CD11c^+^ microglia/macrophage populations from the CL and IL hemispheres of sham-operated WT and KO mice. N = 2 animals. **b.** MFI of pHrodo^+^ cells within CD11b^+^CD45^int^ and CD11b^+^CD45^hi^ populations in the contralateral (CL) and ipsilateral (IL) hemispheres of mice, respectively. Same dataset from **Figure 4b**. **c.** Frequency of p-CREB1^+^ and p-CREB1^+^BDNF^+^ cell counts within parent CD11b^+^CD45^+^ microglia/macrophage populations. **d.** Frequency of p-CREB1^+^ and p-CREB1^+^BDNF^+^ cell counts within parent CD11b^+^CD45^+^ populations (combining CD11b^+^CD45^int^ and CD11b^+^CD45^hi^) . Same dataset from **Figure 5a-b**. * p < 0.05, **** p < 0.0001.

**
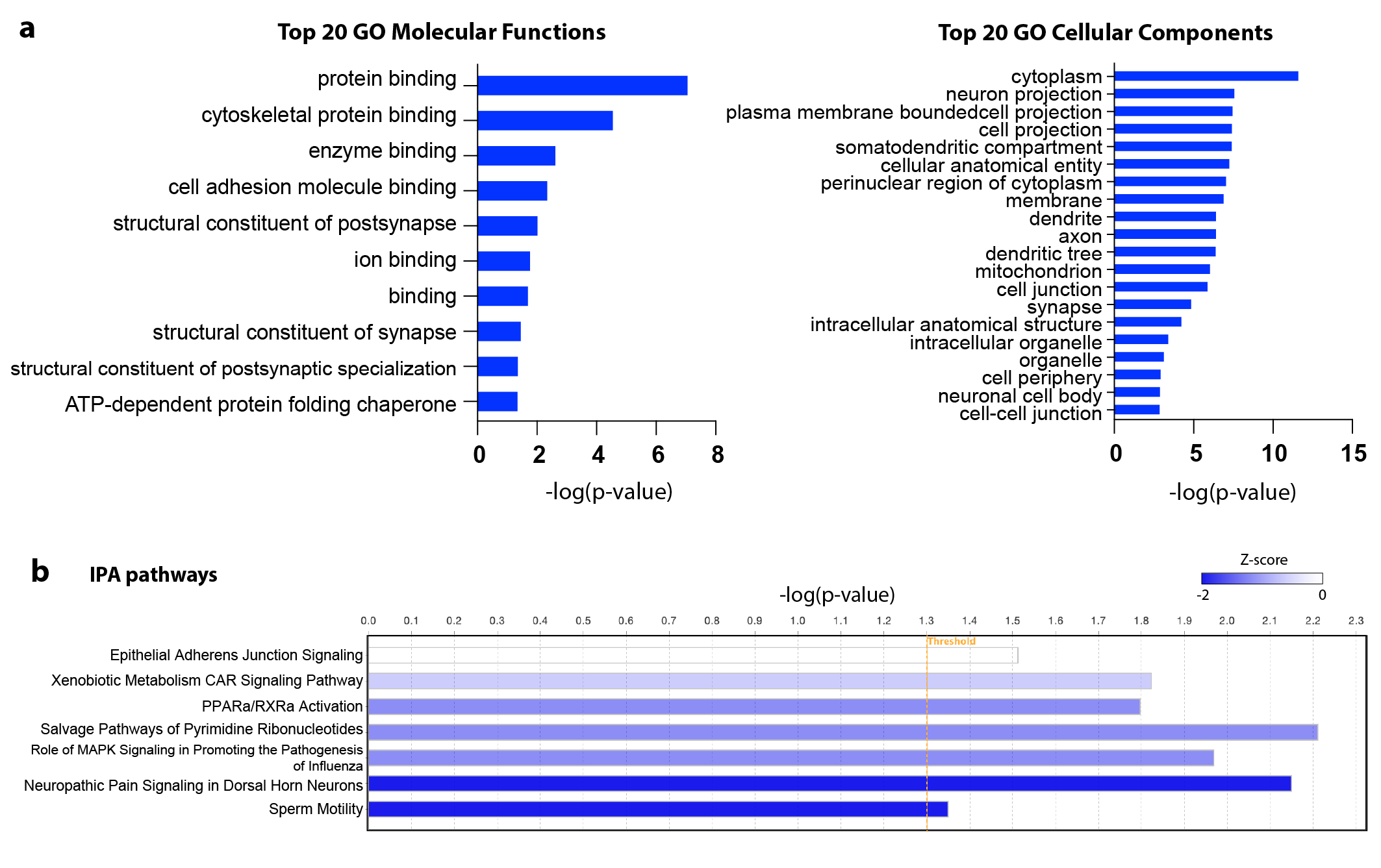
**

**Fig. S5. Transcriptomic pathway analysis with DEGs of OL5 subpopulation.**

**a.** Top 20 molecular functions and cellular components in the Gene Ontology (GO) analysis of OL5 subpopulation from post-stroke white matter tissues (WT and cKO) at 3 d post-tMCAO. **b.** Significantly regulated by the Ingenuity Pathway Analysis (IPA). Gene Set Enrichment Analysis (GSEA), in descending order by z score. Data are from the same dataset as **Figure 1.**

**
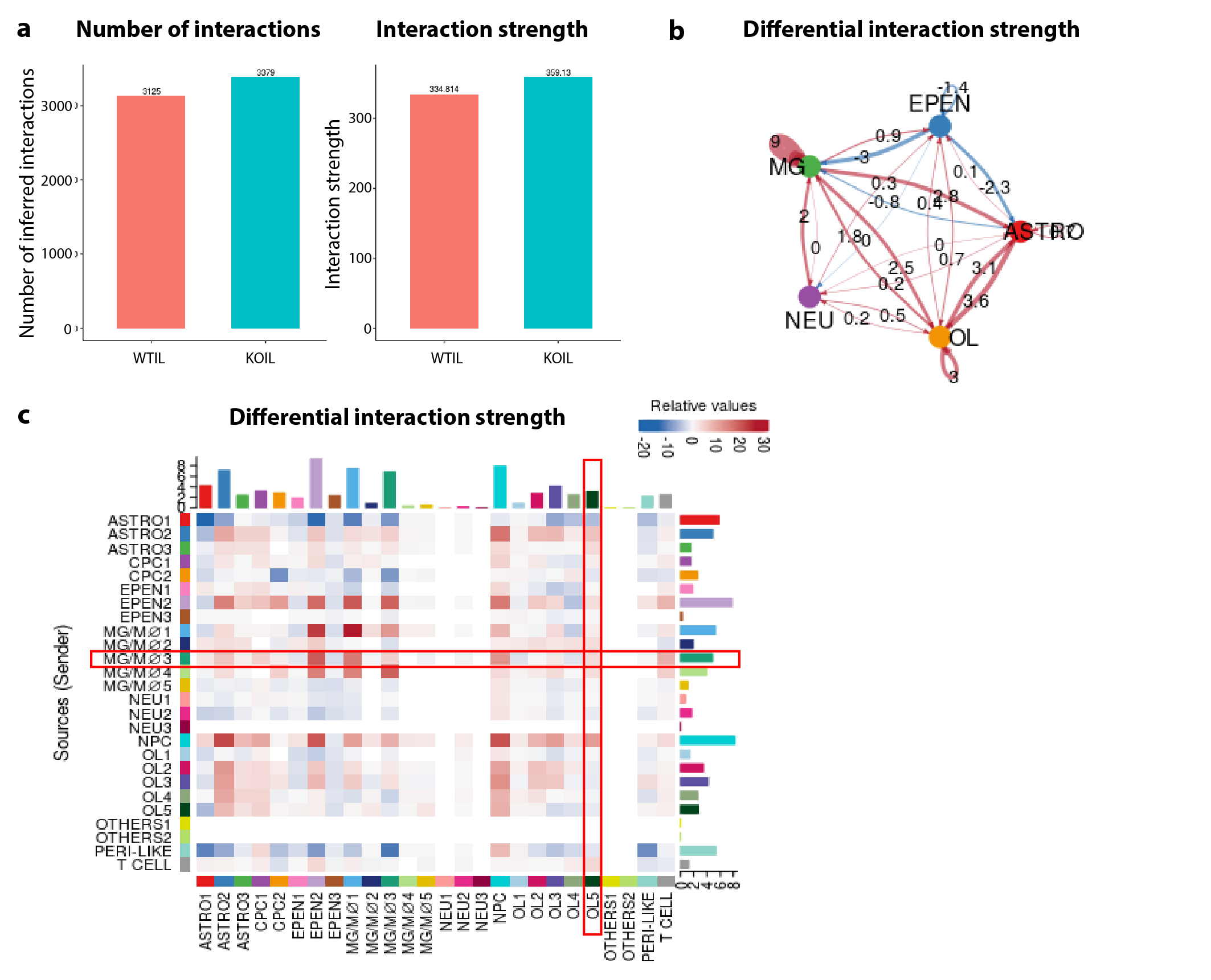
**

**Fig. S6. Cell-cell interactions revealed by CellChat analysis in the *Nhe1* cKO white matter tissues after stroke.**

**a.** Cells in the post-stroke white matter tissues of *Nhe1* cKO brains exhibited overall higher number and strength of cell-cell interactions. **b-c.** Chord diagram and heatmap showing the interaction strength of differential cell-cell interactions between different cell types in the post-stroke white matter tissues of the *Nhe1* cKO brains, compared to WT. Data are from the same dataset as **Figure 1.**


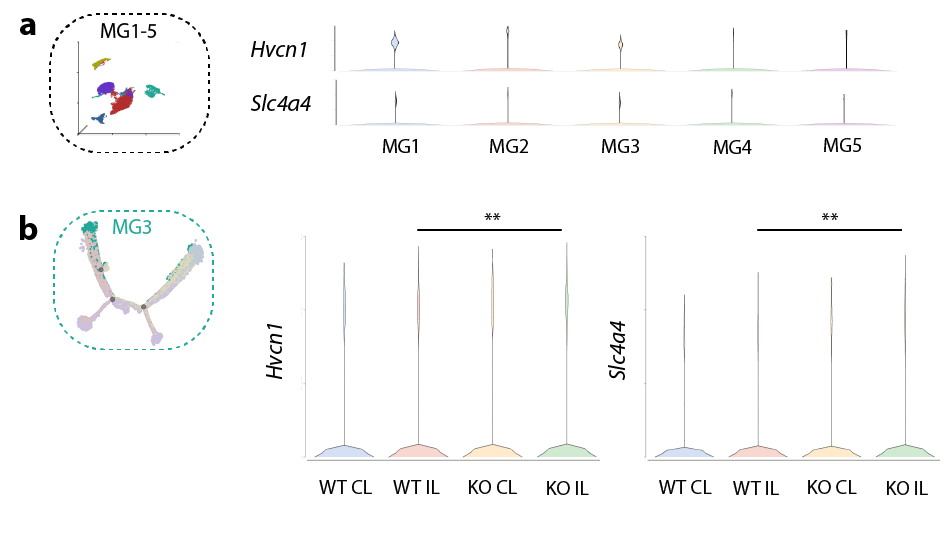


**Fig. S7. Other pH regulating mechanisms in the *Nhe1* cKO white matter tissues after stroke.**

**a.** MG3 exhibited a relatively low expression of *Hvcn1* (encoding Hv1) and *Slc4a4* (encoding NBCe1) comparing to other MG subpopulations (MG1, 2, 4, 5) in post-stroke white matter tissues (WT and cKO) at 3 d post-tMCAO. **b.** *Nhe1* cKO exhibited elevated genes for *Hvcn1* and *Slc4a4* in MG3 subtype in post-stroke white matter tissues (IL hemisphere), compared to WT IL. ** P < 0.01. Data are from the same dataset as **Figure 1.**

**References**

1 Percie du Sert, N. *et al.* The ARRIVE guidelines 2.0: Updated guidelines for reporting animal research. *J Cereb Blood Flow Metab* **40**, 1769-1777, doi:10.1177/0271678X20943823 (2020).

2 Song, S. *et al.* Selective role of Na(+) /H(+) exchanger in Cx3cr1(+) microglial activation, white matter demyelination, and post-stroke function recovery. *Glia* **66**, 2279-2298, doi:10.1002/glia.23456 (2018).

3 Valny, M., Honsa, P., Kirdajova, D., Kamenik, Z. & Anderova, M. Tamoxifen in the Mouse Brain: Implications for Fate-Mapping Studies Using the Tamoxifen-Inducible Cre-loxP System. *Front Cell Neurosci* **10**, 243, doi:10.3389/fncel.2016.00243 (2016).

4 Fogg, D. K. *et al.* A clonogenic bone marrow progenitor specific for macrophages and dendritic cells. *Science* **311**, 83-87, doi:10.1126/science.1117729 (2006).

5 Ajami, B., Bennett, J. L., Krieger, C., Tetzlaff, W. & Rossi, F. M. Local self-renewal can sustain CNS microglia maintenance and function throughout adult life. *Nat Neurosci* **10**, 1538-1543, doi:10.1038/nn2014 (2007).

6 Parkhurst, C. N. *et al.* Microglia promote learning-dependent synapse formation through brain-derived neurotrophic factor. *Cell* **155**, 1596-1609, doi:10.1016/j.cell.2013.11.030 (2013).

7 Hashimoto, D. *et al.* Tissue-resident macrophages self-maintain locally throughout adult life with minimal contribution from circulating monocytes. *Immunity* **38**, 792-804, doi:10.1016/j.immuni.2013.04.004 (2013).

8 Begum, G. *et al.* Selective knockout of astrocytic Na(+) /H(+) exchanger isoform 1 reduces astrogliosis, BBB damage, infarction, and improves neurological function after ischemic stroke. *Glia* **66**, 126-144, doi:10.1002/glia.23232 (2018).

9 Lee, E. *et al.* MPTP-driven NLRP3 inflammasome activation in microglia plays a central role in dopaminergic neurodegeneration. *Cell Death Differ* **26**, 213-228, doi:10.1038/s41418-018-0124-5 (2019).

10 Zhang, B. *et al.* The specificity and role of microglia in epileptogenesis in mouse models of tuberous sclerosis complex. *Epilepsia* **59**, 1796-1806, doi:10.1111/epi.14526 (2018).

11 Schafer, D. P. *et al.* Microglia contribute to circuit defects in Mecp2 null mice independent of microglia-specific loss of Mecp2 expression. *Elife* **5**, doi:10.7554/eLife.15224 (2016).

12 Song, S. *et al.* Activation of endothelial Wnt/beta-catenin signaling by protective astrocytes repairs BBB damage in ischemic stroke. *Prog Neurobiol* **199**, 101963, doi:10.1016/j.pneurobio.2020.101963 (2021).

13 Song, S. *et al.* Elevated microglial oxidative phosphorylation and phagocytosis stimulate post-stroke brain remodeling and cognitive function recovery in mice. *Commun Biol* **5**, 35, doi:10.1038/s42003-021-02984-4 (2022).

14 Jin, S. *et al.* Inference and analysis of cell-cell communication using CellChat. *Nat Commun* **12**, 1088, doi:10.1038/s41467-021-21246-9 (2021).

15 Chen, D. *et al.* Interleukin 13 promotes long-term recovery after ischemic stroke by inhibiting the activation of STAT3. *J Neuroinflammation* **19**, 112, doi:10.1186/s12974-022-02471-5 (2022).

16 McGill, B. E. *et al.* Abnormal Microglia and Enhanced Inflammation-Related Gene Transcription in Mice with Conditional Deletion of Ctcf in Camk2a-Cre-Expressing Neurons. *J Neurosci* **38**, 200-219, doi:10.1523/JNEUROSCI.0936-17.2017 (2018).
